# Self‐Aggregating Tau Fragments Recapitulate Pathologic Phenotypes and Neurotoxicity of Alzheimer's Disease in Mice

**DOI:** 10.1002/advs.202302035

**Published:** 2023-08-18

**Authors:** Ly Thi Huong Luu Le, Jeeyoung Lee, Dongjoon Im, Sunha Park, Kyoung‐Doo Hwang, Jung Hoon Lee, Yanxialei Jiang, Yong‐Seok Lee, Young Ho Suh, Hugh I. Kim, Min Jae Lee

**Affiliations:** ^1^ Department of Biochemistry and Molecular Biology Seoul National University College of Medicine Seoul 03080 South Korea; ^2^ Department of Biomedical Sciences Seoul National University Graduate School Seoul 03080 South Korea; ^3^ Brain Science Institute Korea Institute of Science and Technology Seoul 02792 South Korea; ^4^ Department of Chemistry Korea University Seoul 02841 South Korea; ^5^ Department of Physiology Seoul National University College of Medicine Seoul 03080 South Korea; ^6^ School of Medicine Linyi University Linyi 276000 China; ^7^ Neuroscience Research Institute Seoul National University College of Medicine Seoul 03080 South Korea; ^8^ Ischemic/Hypoxic Disease Institute, Convergence Research Center for Dementia Seoul National University College of Medicine Seoul 03080 South Korea

**Keywords:** aggregation, Alzheimer's disease, axon initial segment, phosphorylation, tau

## Abstract

In tauopathy conditions, such as Alzheimer's disease (AD), highly soluble and natively unfolded tau polymerizes into an insoluble filament; however, the mechanistic details of this process remain unclear. In the brains of AD patients, only a minor segment of tau forms β‐helix‐stacked protofilaments, while its flanking regions form disordered fuzzy coats. Here, it is demonstrated that the tau AD nucleation core (tau‐AC) sufficiently induced self‐aggregation and recruited full‐length tau to filaments. Unexpectedly, phospho‐mimetic forms of tau‐AC (at Ser324 or Ser356) show markedly reduced oligomerization and seeding propensities. Biophysical analysis reveal that the N‐terminus of tau‐AC facilitates the fibrillization kinetics as a nucleation motif, which becomes sterically shielded through phosphorylation‐induced conformational changes in tau‐AC. Tau‐AC oligomers are efficiently internalized into cells via endocytosis and induced endogenous tau aggregation. In primary hippocampal neurons, tau‐AC impaired axon initial segment plasticity upon chronic depolarization and is mislocalized to the somatodendritic compartments. Furthermore, it is observed significantly impaired memory retrieval in mice intrahippocampally injected with tau‐AC fibrils, which corresponds to the neuropathological staining and neuronal loss in the brain. These findings identify tau‐AC species as a key neuropathological driver in AD, suggesting novel strategies for therapeutic intervention.

## Graphical abstract

The study identifies tau nucleation core in Alzheimer's disease (tau‐AC) as a key driver of AD neuropatholgy, which have self‐aggregation and seeding propensities. Tau‐AC oligomers are internalized by cultured cells via endocytosis, leading to endogenous tau aggregation. Tau‐AC aggregates impair axon initial segment (AIS) plasticity in primary neurons and cause abnormal memory retrieval and neuronal loss in mouse brains.

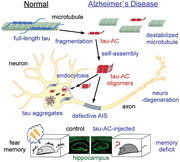

## 1 Introduction

**Figure 1:**
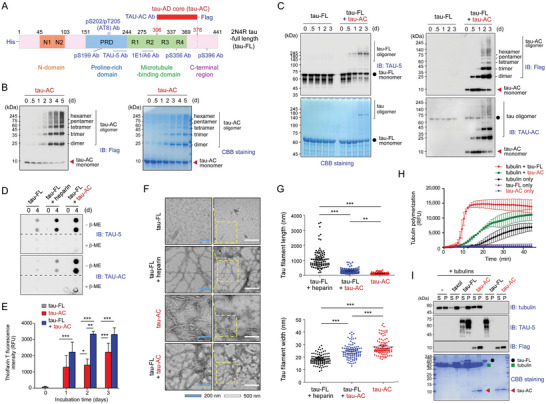
In vitro self‐aggregation and seeding of the Alzheimer's disease tau aggregation core (tau‐AC). A) Schematic diagram depicting the domains of full‐length tau (tau‐FL), tau‐AC (residues 306–378), and locations of antibody epitopes. B) The evaluation of tau‐AC self‐aggregation propensity in the absence of inducers. The FLAG‐tagged recombinant tau‐AC (100 µm) was incubated at 37 °C on a rotating shaker (250 rpm) for indicated time periods and then analyzed using non‐reducing SDS‐PAGE followed by immunoblotting (IB) with anti‐FLAG antibodies (*left*) and Coomassie Brilliant Blue (CBB) staining (*right*). Representative blots from two independent experiments are shown. C) Seeding ability of tau‐AC. Recombinant tau‐FL (20 µm) was incubated alone or with tau‐AC (20 µm) at 37 °C with agitation for indicated time periods. Induction of tau‐FL aggregation by tau‐AC was evaluated by IB using TAU‐5 antibodies and CBB staining (*left*). Self‐aggregation of tau‐AC was detected using anti‐FLAG and TAU‐AC antibodies (*right*). Representative blots from four independent experiments. D) Tau‐FL (20 µm) was incubated with either heparin (80 µm) or tau‐AC (20 µm) for 4 days. The samples were loaded and filtered into each well of a 96‐well plate in the absence or presence of 5% β‐mercaptoethanol (β‐ME). The trapped tau oligomers loaded onto the cellulose acetate membrane were detected by IB using TAU‐5 (for tau‐FL) and TAU‐AC antibodies. E) The samples from the fibrillization reactions performed with 10 μm of tau‐AC and 20 μm of tau‐FL for the indicated time periods were incubated with thioflavin T, and fluorescence intensity at 485 nm was measured. Error bars represent standard errors of the mean. RFU, relative fluorescence unit. ***p < 0.001 (*n* = 4, two‐way ANOVA with the Bonferroni post‐hoc test); n.s., not significant. F) Representative images of negatively stained tau filaments were observed by transmission electron microscopy (TEM). Tau‐FL (20 µm) and tau‐AC (20 µm) were incubated for 2 days at 37 °C. Insets are enlarged images of the enclosed area. White and blue scale bars represent 500 and 200 nm, respectively. G) Quantification of data presented in (F). The length and width of tau filaments were plotted as the mean ± SD. ***p < 0.001 (*n* = 100/group, one‐way ANOVA followed by the Bonferroni post‐hoc test). *p < 0.05, **p < 0.01, ***p < 0.001. H) Tubulin monomers (80 μm) were polymerized in the presence of tau‐FL or tau‐AC (10/1 molar ratio of tubulin/tau) in a GTP‐containing buffer at 37 °C. Microtubule assembly was kinetically monitored by the fluorescence signal from DAPI incorporation into the microtubules (excitation/emission at 358/461 nm; *n* = 3). I) After the microtubule polymerization reaction, the samples were fractionated by ultracentrifugation at 68 000 × *g* for 20 min. Supernatants (S) and pellets (P) were subjected to SDS‐PAGE and analyzed by CBB staining and IB analysis with indicated antibodies.

Filamentous tau inclusions in neurons are the pathological hallmark of a broad class of neurodegenerative diseases collectively called tauopathies. These disorders can be further classified into primary tauopathies, such as corticobasal degeneration (CBD), where tau aggregation is the predominant molecular lesion, and secondary tauopathies, such as Alzheimer's disease (AD), which contain the aggregates of additional proteins.^[^ ^1^ ^]^ Microtubule‐associated protein tau is encoded by *MAPT* gene, generating multiple isoforms via alternative splicing.^[^ ^2^ ^]^ The longest tau isoform (2N4R, 441 residues) contains four functional domains: an N‐domain, proline‐rich domain, microtubule‐binding domain (MBD), and C‐terminal region (**Figure** **1**A), resulting in a highly soluble (average hydropathicity of −0.87) and positively charged (net pI = 8.2) protein.^[^ ^3^ ^]^ All four repeats (R1–R4) in the MBD were initially believed to form the anti‐parallel C‐shaped stacks of β‐sheets in paired helical filaments (PHFs);^[^ ^4^ ^]^ however, recent cryo‐electron microscopy (cryo‐EM) studies showed that insoluble tau filaments can have diverse folding structures depending on the type of tauopathy. For example, tau filaments from the AD brain (consisting of both 3R and 4R isoforms) contained only R3, R4, and a part of the C‐terminal region, distinct from those from Pick's disease (PiD; 3R tauopathy) and CBD (4R tauopathy).^[^ ^5^ ^]^ Furthermore, the recombinant tau fragments, consisting of 2N4R tau residues 306–378 (hereafter named tau‐AC), spontaneously assembled and further induced endogenous tau aggregation.^[^ ^6^ ^]^ However, the underlying mechanisms driving this phenomenon and its pathophysiological implications in AD remain largely uncharacterized.

Recent evidence indicates that post‐translational modifications (PTMs) are critical in the self‐aggregation of otherwise soluble tau.^[^ ^7^ ^]^ Phosphorylation has been considered the major event in tau fibrillization, and more than 85 phosphorylatable residues have been reported in 2N4R tau (≈19% of all amino acids;^[^ ^8^ ^]^ however, no therapeutic effects were observed in clinical trials of drug candidates that were developed based on this premise.^[^ ^9^ ^]^ Furthermore, the inter‐filament packing of some phosphorylated residues of tau‐AC was stabilized by numerous polyubiquitin chains connected to Lys residues, which could provide additional interfacial contacts.^[^ ^5d^ ^]^ Thus, tau phosphorylation might induce its ubiquitylation for proteasomal degradation rather than aggregation at the early AD stage, eventually leading to tau hyperubiquitylation and the formation of insoluble protofilaments under pathological circumstances.^[^ ^3^ ^]^ Tau acetylation, which potentially competes with ubiquitylation at the identical Lys residues, was also found to augment pathologic tau aggregation.^[^ ^10^ ^]^ Therefore, the pathological consequences of tau PTMs need to be further investigated.

Tau proteins are predominantly located in the axons of mature neurons, contributing to microtubule stabilization and axonal transport, as well as synaptic plasticity and neuronal excitability.^[^ ^11^ ^]^ In AD, neurons lose their tau polarization, leading to tau accumulation in the soma and dendrites.^[^ ^12^ ^]^ Pathologic forms of tau detached from microtubules are transported to somatodendritic compartments through the axon initial segment (AIS) located near the hillock of axons with 20–60 µm length. The AIS serves as a diffusion barrier and a selective filter, which sorts the axonal and somatodendritic proteins.^[^ ^13^ ^]^ The AIS cytoskeleton consists of submembrane proteins, cytoskeletal proteins, and filamentous actin (F‐actin).^[^ ^14^ ^]^ The AIS is also critical for neuronal excitability, since it is enriched with voltage‐gated ion channels and initiates axon potential (AP) firing.^[^ ^15^ ^]^ In addition, the anatomical characteristics of the AIS, including its position and length, are dynamically changed under physiological and pathological conditions.^[^ ^16^ ^]^ Pathologic tau localize to dendritic spines to a much greater extent than to dendritic shafts through a direct interaction with F‐actin and affects the AIS cytoskeleton and plasticity, which has been implicated in AD and other tauopathies.^[^ ^17^ ^]^

Given that the intracellular events leading to tau fibrillization remain unclear, identifying the pathological forms of tau species is critical. In this study, we demonstrated that tau‐AC efficiently self‐assembled and triggered the co‐aggregation of full‐length tau. Using biophysical analysis, we identified the exposure of N‐terminal hydrophobic residues of tau‐AC as a potential mechanism of its aggregation propensity. Oligomeric tau‐AC was internalized by cultured cells and primary hippocampal neurons, primarily via caveolin‐mediated endocytosis, triggering endogenous tau aggregation. Isolated primary neurons infected with tau‐AC‐expressing lentiviruses demonstrated a significantly shortened AIS in response to chronic depolarization and mislocalized tau in dendritic spines. Finally, we stereotaxically injected tau‐AC into the mouse hippocampus and found that these mice had significantly increased levels of histopathological staining, altered anxiety‐like behavior, and impaired contextual fear memory retrieval. Collectively, our findings provide an insight into the causal molecular mechanisms of tau aggregation, which could be used for developing novel diagnostic and therapeutic tools.

## 2 Results

### 2.1 Self‐Assembling and Seeding Properties of Tau‐AC In Vitro

To investigate the self‐aggregation and seeding properties of tau‐AC, we first purified the full‐length 2N4R human tau (tau‐FL), as well as its truncated form containing amino acids 306 to 378 (tau‐AC) (Figure 1A). Tau‐AC gradually generated high‐molecular weight species after ≈24 h incubation in different buffers without anionic cofactors, such as heparin or dextran sulfate (Figure 1B; Figure S1A, Supporting Information). When co‐incubated with tau‐FL, tau‐AC induced the oligomerization of tau‐FL, as demonstrated by immunoblotting analysis using TAU‐5 and TAU‐AC antibodies specifically targeting tau‐FL and tau‐AC, respectively (Figure 1C). In the absence of tau‐AC, tau‐FL did not generate any oligomeric species for up to 3 days. We also observed the formation of heterotypic oligomers consisting of both tau‐FL and tau‐AC (Figure S1B, Supporting Information). A strong seeding effect of tau‐AC as apparent when equimolar tau‐AC and tau‐FL were co‐incubated for 2 days while a 0.5:1 or lower molar ratios of tau‐AC to tau‐FL resulted in only minor aggregation of tau‐FL (Figure S1C and S1D, Supporting Information). To directly evaluate tau fibrillization, we performed a filter trap assay and observed that highly soluble tau‐FL became trapped in the membrane when pre‐incubated with tau‐AC (Figure 1D). The insoluble and oligomeric tau‐FL species were also retrieved from the pellet fractions of ultracentrifuged samples that were pre‐incubated with tau‐AC (Figure S1E, Supporting Information).

Subsequently, to monitor the assembly process, we used thioflavin T (ThT), a fluorescent molecular rotor that emits strong fluorescent signals when its C─C bond rotation is inhibited by binding to amyloid fibrils.^[^ ^18^ ^]^ ThT intensity gradually increased with tau‐FL co‐incubated with tau‐AC; however, the signal was virtually absent in samples containing only tau‐FL (Figure 1E). Tau‐AC alone generated stronger ThT signal than that from the same concentration of tau‐FL with heparin after 1 or 2 days of aggregation reactions (Figure S1F, Supporting Information), further indicating its prominent self‐aggregation ability. To verify these findings, samples were evaluated by negative‐staining transmission electron microscopy (TEM) at the 48 h time point (Figure 1F). Unlike tau‐FL, which did not generate fibrillar structures in the absence of heparin, tau‐AC spontaneously formed distinct fibrils. The morphology of tau‐AC fibrils was significantly shorter and thicker than the filament bundles of heparin‐induced tau‐FL (Figure 1G). The short and thick fibrils of tau‐AC could function as effective protofilamental nucleates for further self‐aggregation and recruitment of endogenous tau‐FL into the fibrils. Next, we assessed the effect of tau‐AC on microtubule assembly using tubulin oligomerization and sedimentation assays. We observed that tau‐FL effectively stabilized microtubules; however, in a sharp contrast, tau‐AC showed only a minor enhancement of microtubule assembly and displayed considerably weaker binding to tubulin compared to tau‐FL (Figure 1H,I), which is consistent with a recent report identifying that Lys369–Lys395 are the microtubule‐binding residues in tau.^[^ ^19^ ^]^ Collectively, these data suggest that tau‐AC is less associated than tau‐FL with microtubules and has an intrinsic ability to form pathologic aggregates in vitro even without aggregation inducers or PTMs.

### 2.2 Antagonistic Effects of Tau‐AC Phosphorylation on the Self‐Aggregation and Seeding

**Figure 2:**
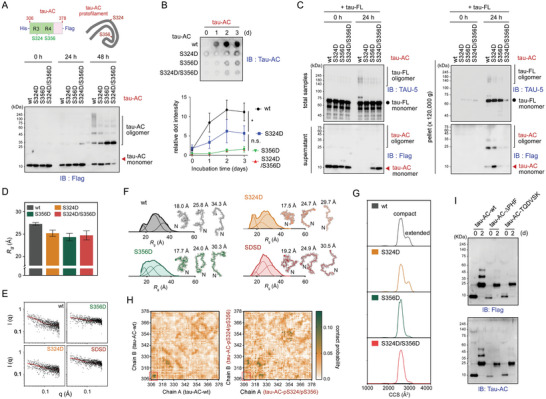
Biochemical and biophysical analysis of tau‐AC self‐aggregation. A) Serines at 324 and 356 positions of tau‐AC were mutated to aspartic acids to mimic its phosphorylation (tau‐AC‐S324D, ‐S356D, and ‐S324D/S356D [SDSD] mutants; *top*). Wild‐type tau‐AC (tau‐AC‐wt) or phospho‐mimetic mutants (phospho‐tau‐AC; 40 µm each) were incubated at 37 °C for indicated time periods. Samples were subjected to non‐reducing SDS‐PAGE/IB (*bottom*). Representative blots from four independent experiments. B) The conversion of tau‐AC into insoluble aggregates was analyzed by filter trap assays over a 3‐day period. Trapped tau fibrils were detected by IB using TAU‐AC antibodies (*top*), quantified, and plotted as the mean ± SD (*bottom*); results from three independent experiments (*p < 0.05, two‐way ANOVA using the Bonferroni post‐hoc test); n.s., not significant. C) The seeding propensity of phospho‐tau‐AC. Tau‐FL (20 µm) was incubated with tau‐AC or phospho‐tau‐AC (20 µm) at 37 °C for 24 h, and the assembly reactions were ultracentrifuged at 120 000× *g* for 1 h to obtain supernatant and pellet fractions. The fractions were analyzed by SDS‐PAGE/IB using anti‐FLAG (for tau‐AC) and TAU‐5 (for tau‐FL) antibodies. Representative blots from two independent experiments. D) The radius of gyration (*R_g_*) values and *R_g_* distribution of tau‐AC‐wt and tau‐AC‐S324D, ‐S356D, and ‐SDSD mutants were obtained from the Guinier analysis of small‐angle X‐ray scattering (SAXS) profiles. The error bars represent the standard deviation of two independent SAXS experiments. E) Raw X‐ray scattering patterns with experimental SAXS profiles (black dots) and fitted curves (red lines) obtained from the ensemble optimization method (EOM) from tau‐AC‐wt and three phospho‐mimetic mutants. F) Deconvoluted *R_g_* distribution from the EOM analysis and the representative structures of each tau‐AC species. G) Collision cross‐section (CCS) distribution of tau‐AC‐wt and the mutants. H) Interchain contact probability maps of tau‐AC‐wt and its double phosphorylated form (pS324/pS356) from 30.4 µs molecular dynamics simulation trajectory. Red boxes indicate the ^306^VQIVYK^311^ hexapeptides located at the N‐termini of tau‐AC. I) Self‐aggregation of the tau‐AC lacking the intact PHF6 motif. The experiments were performed as described in Figure 1B, with tau‐AC mutants being used instead of tau‐AC‐wt. Representative blots from two independent experiments.

The majority of tau phosphorylation occurs at the residues located outside of the MBD and this is considered to be an early pathologic event in AD.^[^ ^20^ ^]^ Using an in vitro phosphorylation reaction and MS analysis, we identified two phosphorylation sites, Ser324 and Ser356, in tau‐AC; both sites contained the Lys‐Ile‐Gly‐Ser motif in the R3 and R4 repeats, respectively (Figure S2A, Supporting Information).^[^ ^3^ ^]^ Unexpectedly, tau‐AC, which was phosphorylated with recombinant GSK3β, showed considerably less self‐aggregation than intact tau‐AC (Figure S2B, Supporting Information). To further investigate the consequences of site‐specific phosphorylation, we purified wild‐type tau‐AC (tau‐AC‐wt) and its phospho‐mimetic point mutants (phospho‐tau‐AC; S324D, S356D, and S324D/S356D [SDSD]), and performed the in vitro self‐assembly assay. Unlike aggregation‐prone tau‐AC‐wt, the phospho‐tau‐AC species appeared to form dimers primarily, demonstrating a significantly reduced level of high‐molecular‐weight oligomers (**Figure** **2**A). Furthermore, tau‐AC‐S356D had a more prominent decrease in oligomerization than did tau‐AC‐S324D. Consistent with the results from gel‐based assays, the ThT and filter trap assays demonstrated that pseudo‐phosphorylation mutants, especially tau‐AC‐S356D, exhibited drastically reduced self‐aggregation (Figure 2B; Figure S2C and S2D, Supporting Information). In addition, TEM images showed that the mutants appeared to generate smaller number of fibrils than did tau‐AC‐wt (Figure S2E, Supporting Information). After ultracentrifugation, we found oligomeric forms of tau‐FL in the pellet fraction with tau‐AC‐wt, but in less extent with its phospho‐mimetic mutants (Figure 2C). Tau‐AC‐SDSD drastically reduced the levels of total tau‐FL in the pellet fraction, strongly suggesting that phosphorylation might interfere with the seeding ability of tau‐AC. These results indicate that tau‐AC phosphorylation may affect its fibrillization kinetics and have an antagonistic effect on tau aggregation.

To investigate the mechanism underlying the biochemical properties of tau‐AC species, we performed structural analysis using small‐angle X‐ray scattering (SAXS). The Kratky analysis of the SAXS profiles and circular dichroism (CD) spectra indicated that both wild‐types and phospho‐mimetic mutants of tau‐AC were largely unstructured and random‐coiled (Figure S3A and S3B, Supporting Information). The experimental radius of gyration (*R*_g_) values from the SAXS profiles indicated that the relatively extended tau‐AC‐wt (27.25 ± 0.30 Å) became more compact after phosphorylation (25.20 ± 0.70, 24.33 ± 0.84, and 24.72 ± 1.01 Å for tau‐AC‐S324D, ‐S356D, and ‐SDSD mutants, respectively) (Figure 2D). Next, we performed the ensemble optimization method (EOM) analysis of the X‐ray scattering patterns, combined with replica‐exchange molecular dynamics (REMD) simulations. The resulting conformation pool of each tau‐AC species revealed that the phospho‐mimetic mutants had a more compact structure than tau‐AC‐wt, confirming the results of *R*_g_ analysis (Figure 2E,F). The extended conformer distribution of tau‐AC‐wt was centered at 34.52 Å, while the distribution of pseudo‐phosphorylation mutants was 28.93, 30.11, and 31.00 Å for S324D, S356D, and SDSD, respectively. We also found that the N‐terminal residues of tau‐AC containing the hexapeptide motif ^306^VQIVYK^311^ (also known as the PHF6 motif)^[^ ^21^ ^]^ had the lowest solubility scores (< −1) (Figure S3C, Supporting Information). Our calculated conformations suggested that the PHF6 motifs of tau‐AC‐wt and tau‐AC‐S324D were highly accessible for oligomerization; in contrast, those of tau‐AC‐S356 and tau‐AC‐SDSD mutants were largely sterically shielded (Figure 2F). This conformational change is accompanied by an increase in the intramolecular contact probability within the N‐terminal residues: quantitative analysis of fifty ensemble structures revealed that the distance between the N‐ and C‐terminus is 7.63 ± 0.37 nm in the tau‐AC‐wt and 5.91 ± 0.41, 6.43 ± 0.40, and 6.95 ± 0.34 nm in the S324D, S356D, and S324D/S356D variants, respectively (Figure S3D, Supporting Information). Taken together, these results implied that the experimentally observed compaction in terms of radius of gyration might be due to the shortened distance between the N‐ and C‐terminal residues in solution. Thus, we postulated that the exposed N‐terminal PHF motif might function as the critical contact point for the self‐aggregation of tau‐AC. In addition, tau‐AC phosphorylation might induce a conformational change through enhanced intramolecular electrostatic interactions, bringing the N‐termini inward and obstructing their interactions.

The structural properties of tau‐AC and its phospho‐mimetic mutants were further analyzed using electrospray ionization/ion mobility mass spectrometry (ESI‐IM‐MS). The IM spectra also indicated that tau‐AC‐wt and ‐S324D had an extra population with extended conformations, whereas tau‐AC‐S356D and ‐SDSD mutants only showed compact conformations (Figure 2G; Figure S3E, Supporting Information). To gain a mechanistic insight into the interpeptide interactions, next, we performed an *in silico* analysis of tau‐AC dimers by running all‐atom REMD simulation sets consisting of 76 different temperatures, ranging from 300 to 400 K with equal exchange probabilities. The obtained contact maps from equilibrated trajectories revealed that tau‐AC‐wt retained strong dimeric interactions via the ^306^VQIVYK^311^ hexapeptide motifs at the N‐terminus, while the double phosphorylated tau‐AC (tau‐AC‐pS324/pS356) showed significantly weakened interchain interactions (Figure 2H; Figure S3F, Supporting Information). The contact probability maps of tau‐AC‐pS324/pS356, which was not self‐aggregated (Figure 2H), showed global differences from tau‐AC‐wt, indicating a significant change in the 3D structures induced by tau‐AC phosphorylation. Tau‐AC‐pS324 exhibited a relatively higher probability of the interchain contact through the N‐terminal hexapeptide motifs, compared to tau‐AC‐pS356 and ‐pS324/pS356 (Figure S3G and S3H, Supporting Information). Finally, we carried out in vitro aggregation reactions with other tau‐AC mutants, which lacked the PHF6 motif (tau‐AC‐ΔPHF6) and were substituted with hydrophilic residues (tau‐AC‐TQDVSK). Unlike tau‐AC‐wt, these mutants failed to oligomerize to higher than dimeric forms and did not induce tau‐FL aggregation (Figure 2I; Figure S4A–S4D, Supporting Information). Therefore, the hydrophobic interactions between the N‐terminal motifs appeared to play a nucleating role at the early stage of tau‐AC aggregation. Moreover, our integrated biophysical and biochemical data suggested that phosphorylation‐mediated structural changes from extended to compact forms could interfere with the interactions between the PHF6 motifs.

### 2.3 Cellular Uptake of Tau‐AC Aggregates and Its Effect on Endogenous Tau

**Figure 3:**
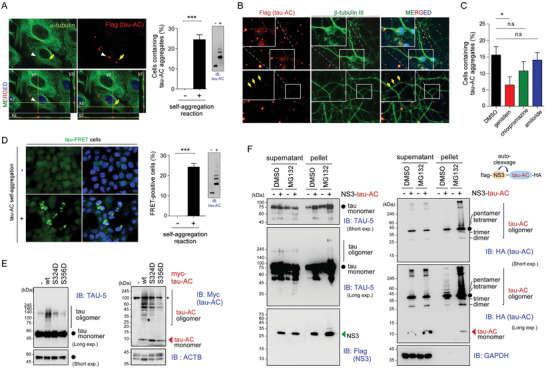
Cellular uptake of tau‐AC aggregates by cell lines and primary neurons. A) (*left*) Representative confocal images of A549 cells cultured with tau‐AC^FLAG^ (1 μm final concentration) before (–) and after (+) self‐aggregation reactions for 24 h. Cells were trypsinized, re‐plated onto the new glass coverslips, and then stained with anti‐FLAG and anti‐α‐tubulin antibodies. Internalized tau‐AC aggregates were imaged as Z‐stacks (X‐Z and Y‐Z cross sections). (*right*) The percentage of tau‐AC‐positive cells in the whole population were quantified and presented as the mean ± SD of three independent experiments. ***p < 0.001 (*n* > 100 cells per group, two‐tailed unpaired Student's *t‐*test). The tau‐AC samples were also analyzed by IB using TAU‐AC antibodies. B) Day 10 in vitro (DIV 10) primary rat hippocampal neurons were treated with self‐aggregated tau‐AC (0.1 µm) for 3 days, vigorously washed three times, and then co‐immunostained using anti‐β‐tubulin III/Tuj‐1 (for differentiating neurons) and anti‐FLAG (for tau‐AC) antibodies. Nuclei were counterstained with DAPI. Yellow arrows indicate intraneuronal tau‐AC. C) A549 cells were pre‐treated for 1 h with endocytosis inhibitors genistein (200 µm), chlorpromazine (20 µm), and amiloride (500 µm), incubated with oligomeric tau‐AC for 24 h, and then the percentage of tau‐AC‐positive cells were quantified; *p < 0.01 (*n* > 100 cells per group, one‐way ANOVA followed by the Bonferroni post‐hoc test); n.s., not significant. D) Förster resonance energy transfer (FRET)‐based analysis of tau‐AC seeding effects in HEK293 tau‐P301S biosensor cells. The cells were treated with 1 μm of tau‐AC before (–) and after (+) self‐aggregation for 24 h, and a relative FRET‐positive cell number was quantified. To detect the FRET signal, cells were excited with a 488 nm laser and fluorescence was captured using a 525/50 filter. Data are represented as the mean ± SD; ***p < 0.001 (*n* > 500 cells/group, two‐tailed unpaired Student's *t‐*test). E) Primary cortical rat neurons were infected with lentiviruses expressing Myc‐tagged tau‐AC and its phospho‐mimetic mutants on DIV 6. Whole‐neuron lysates were collected on DIV 13 and subjected to non‐reducing SDS‐PAGE/IB. *, non‐specific signals. F) HA‐tagged tau‐AC was fused with NS3 protease containing an auto‐cleavage site located at the junction between NS3 and tau‐AC. HEK293T cells were co‐transfected with plasmids expressing NS3‐tau‐AC and tau‐FL for 30 h and then treated with MG132 (10 μm) for additional 6 h. Cells were lysed in RIPA buffer, whole cell lysates were separated into supernatant and pellet fractions and subjected to non‐reducing (supernatants) and reducing (pellets) SDS‐PAGE/IB. Representative blots from two independent experiments.

Next, we evaluated whether the spontaneously aggregated tau‐AC species could be internalized by cells in a manner similar to other aggregation‐prone proteins, such as amyloid β peptides, prion proteins, and huntingtin with expanded polyglutamine repeats.^[^ ^22^ ^]^ A549 cells and primary hippocampal neurons were incubated with FLAG‐tagged monomeric tau‐AC (no self‐assembly reaction) or oligomeric tau‐AC (2‐day self‐oligomerization reaction), vigorously washed, trypsinized to completely remove the membrane‐bound tau‐AC, and replated. Immunostaining using anti‐FLAG antibodies demonstrated that intracellular inclusions were co‐localized with α‐tubulin and DAPI (**Figure** **3**A; Figure S5A, Supporting Information). We also observed that internalized tau‐AC puncta were found in ≈25% of cells treated with oligomeric tau‐AC; however, no tau‐AC‐positive inclusions were detected in monomeric tau‐AC‐treated cells (Figure 3A). In primary neurons, most tau‐AC signals were observed in the soma, while a small number of punctate signals was also detected in the axons (Figure 3B). Tau‐AC aggregates were found primarily localized in the endosomes and lysosomes, as identified by co‐immunostaining with Rab5, Rab7, and LAMP1 antibodies (Figure S5B, Supporting Information). Because the majority of intracellular tau‐AC appeared to be trafficked to the downstream compartments of the endocytic transport system, we tested several endocytosis inhibitors. Our results showed that genistein (caveolin‐mediated endocytosis inhibitor) reduced tau‐AC internalization, while amiloride (a macropinocytosis inhibitor) had minimal effect (Figure 3C). Chlopromazine, a clathrin‐mediated endocytosis inhibitor, also decreased the cellular uptake of tau‐AC, but to a lesser degree. These data suggested that the oligomeric species of tau‐AC, but not its monomeric form, are actively internalized through caveolin‐mediated endocytosis, rather than passive transmembrane diffusion.

Next, to examine whether internalized tau‐AC stimulated the aggregation of tau‐FL, we used HEK293‐tau‐P301S‐biosensor (tau‐FRET) cells, ^[^ ^23^ ^]^ which generate Förster resonance energy transfer (FRET) signals in response to tau‐CFP and ‐YFP polymerization. Upon the exposure of the tau‐FRET cells to oligomeric forms of tau‐AC, the FRET signal was considerably increased compared to the negligible background signal (Figure 3D). In addition, there was a direct correlation between the FRET signal and the amount of tau‐AC aggregates that the tau‐FRET cells were incubated with (Figure S5C, Supporting Information). The FRET signal was not detected in cells incubated with monomeric tau‐AC. These findings indicated that the seeding ability of tau‐AC selectively corresponded to the pathological aggregates in live cells. Next, we overexpressed tau‐AC‐wt in primary neurons using lentiviruses and observed that the level of tau oligomers was significantly increased in a gel‐based assay (Figure 3E). To further confirm these findings, we cloned tau‐AC into a vector that included an N‐terminally fused NS3 protease, which yields tau‐AC lacking the N‐terminal Met (start codon) residue (Figure 3F). Upon transient overexpression of this construct in the presence of proteasome inhibitors, the high‐molecular weight species of both tau‐FL and tau‐AC were observed in the pellet fractions of whole‐cell lysates (Figure 3F), providing further evidence of tau‐AC seeding ability.

Next, we investigated the effect of tau‐AC phosphorylation on endogenous tau aggregation using tau‐FRET cells. Virtually no FRET signal or tau puncta were detected in cells treated with tau‐AC‐S324D, ‐S356D, or ‐SDSD, unlike in tau‐AC‐wt‐treated cells. This absence of signals could be attributed to the weak self‐aggregation propensities of the phospho‐mimetic mutants (Figure S5D, Supporting Information). Primary cortical neurons infected with lentiviruses expressing phospho‐mimetic forms of tau‐AC generated significantly lesser oligomeric forms of endogenous tau‐FL than those in cells expressing tau‐AC‐wt (Figure 3E). Similarly, A549 cells treated with tau‐AC‐S324D and ‐S356D produced noticeably lesser punctate or aggregate signals than that produced by cells incubated with tau‐AC‐wt (Figure S5E, Supporting Information). Taken together, our cell studies largely recapitulated the results of the in vitro experiments demonstrating the seeding ability of tau‐AC as well as the negative effect of its phosphorylation on self‐fibrillization.

### 2.4 Tau‐AC Impaired AIS Plasticity and Polarized Trafficking in Neurons

**Figure 4:**
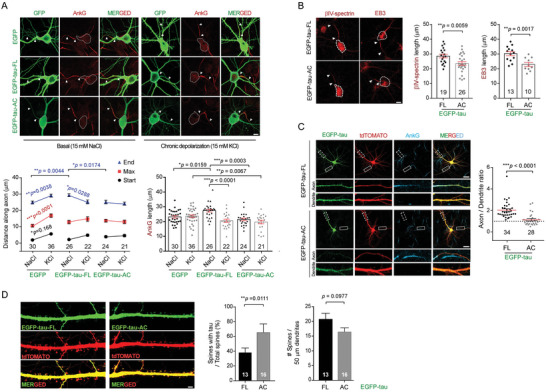
Impaired AIS plasticity in primary hippocampal neurons overexpressing tau‐AC. A) Shortened AIS length in tau‐AC‐transfected neurons during chronic depolarization. EGFP‐tagged tau‐FL and tau‐AC were transiently overexpressed in DIV 10 primary hippocampal neurons. On DIV 12, the infected neurons were further treated with either 15 mm of NaCl (as controls) or KCl (for depolarization) for another 48 h. Representative confocal images (*top*) and quantification of AIS localization and length (*bottom*) are shown. The start and end points of the AIS detected by ankyrin G (AnkG) immunostaining are indicated with white arrowheads. Distances along the axon and AIS length were analyzed using two‐way ANOVA and one‐way ANOVA, respectively, followed by Tukey multiple comparison test. The number of neurons used for each quantification is shown at the bottom of the graph. Scale bar, 10 µm. B) The experiments were performed as described in (A), except that βIV‐spectrin and EB3 immunostaining was performed (*left*), AIS lengths were quantified (*right*), and data were analyzed using the unpaired Student's *t*‐test. The boundary of soma is shown with a white dashed line. Scale bar, 10 µm. C) Polarized trafficking is affected in neurons overexpressing tau‐AC. DIV 14 hippocampal neurons were co‐transfected with EGFP‐tau‐FL or ‐AC and tdTOMATO. After 24 h, cells were immunostained with anti‐AnkG antibody and Z‐stacked confocal microscopy images were acquired. Using the MetaMorph software, axonal and dendritic tau signals were quantified using the AnkG‐positive axon and soma‐excluded dendritic branches, respectively. Dotted‐line square, axons (20 µm from the soma). Solid‐line square, dendrites (50 µm from the soma). The results were normalized by axon to dendritic tdTOMATO signal intensities, which were obtained using the same method. Arrowheads indicate AnkG‐positive axons. ***p < 0.0001 (two‐tailed unpaired Student's *t*‐test). Scale bar, 25 µm. D) Effect of tau‐AC on dendritic spine density and tau localization in primary rat neurons. EGFP‐tau‐FL and ‐AC were overexpressed in DIV 13 neurons, and fluorescence signals were obtained using Z‐stacked confocal microscopy on DIV 21. Representative images and spine quantification are shown. Tau‐positive spines were determined using the semi‐automated NeuronJ program. Bars represent the percentage of tau‐positive dendritic spines and the number of total spines (mean ± SD; two‐tailed unpaired Student's *t*‐test). Scale bar, 5 µm.

Because tau‐AC was unable to strongly bind to tubulin but interacted with end‐binding protein 3 (EB3), a structural component of the AIS (Figure S5F, Supporting Information), we hypothesized that overexpression of tau‐AC would have a dominant‐negative effect on microtubule dynamics and AIS plasticity. First, we examined differences in the localization and length of the AIS in response to changes in neuronal activity. Dissociated hippocampal neurons were treated with 15 mm extracellular potassium ion (K^+^) from days in vitro (DIV) 12 to 14, and the AIS was immunostained for the essential AIS scaffolding protein ankyrin G (AnkG). As reported previously,^[^ ^24^ ^]^ chronic depolarization using K^+^ relocated the AIS distally 4–5 µm down the axon; however, all start, maximum, and end positions were simultaneously shifted, leaving the AIS length unchanged (**Figure** **4**A). The primary function of the AIS relocation is to modulate neuronal excitability and AP spike threshold.^[^ ^24^ ^]^ In response to the overexpression of tau‐FL under basal conditions, we observed a significant distal shift of the AIS end positions but no changes in start and maximum positions, resulting in increased AIS length (Figure 4A). The lengthening of AIS by tau‐FL was effectively reversed upon chronic depolarization by proximal translocation of the AIS end points, suggesting that neurons expressing tau‐FL partially mimicked the effect of hyperphosphorylated tau‐E14 expression in steady–states^[^ ^25^ ^]^ but still retained activity‐dependent AIS plasticity. In contrast, neurons overexpressing tau‐AC did not respond to the prolonged K^+^ signal, showing little changes in the AIS position (Figure 4A). By monitoring additional AIS markers EB3 and βIV‐spectrin, we observed a similar result: a significant shortening of the AIS length in response to tau‐AC (Figure 4B). These data indicated that the AIS plasticity was severely impaired by tau‐AC, providing a possible explanation for neuronal network hyperexcitability and excitotoxicity observed in various AD mouse models.^[^ ^26^ ^]^ To further elucidate the molecular mechanism involving tau‐AC and AIS structural plasticity, we treated the neurons with a low dose (1 nm) of the microtubule‐stabilizing agent paclitaxel for 3 h. Consistent with a previous report,^[^ ^27^ ^]^ we observed a significant increase in AIS length, characterized by a distal shift in AIS end position, similar to the effect of tau‐FL (Figure S5G, Supporting Information). There were no synthetic effects between tau‐FL overexpression and paclitaxel treatment. In sharp contrast, tau‐AC‐wt effectively attenuated the AIS lengthening effect induced by paclitaxel, but its phospho‐mimetic tau‐AC‐SDSD mutant did not (Figure S5G, Supporting Information). These findings suggest that AIS structural plasticity is dysregulated due to the self‐aggregation of tau‐AC rather than the reduced affinity of tau‐AC toward tubulins.

Next, to determine whether AIS plasticity regulates the polarized trafficking of tau from the soma to axon, we investigated the localization of overexpressed tau‐AC in primary neurons. We co‐transfected hippocampal neurons with either EGFP‐tagged tau‐FL or tau‐AC and tandem dimer Tomato (tdTomato), which was used to normalize the axon‐to‐dendrite distribution (as it is uniformly distributed across axons and dendrites). We observed that overexpressed tau‐FL was distributed throughout the neurons; however, the tdTomato‐normalized axon‐to‐dendrite ratio was ≈2, indicating that tau‐FL was preferentially localized in axons rather than dendritic branches (Figure 4C). In contrast, a significant fraction of tau‐AC was mislocalized to the somatodendritic compartment (Figure 4C), suggesting that the selective filtering function of the AIS was disrupted by overexpressed tau‐AC. We also evaluated whether mislocalized tau‐AC changed the synaptic function by affecting the number of dendritic spines. Tau‐AC and tdTomato double‐labeling showed that tau‐AC only modestly decreased the number of dendritic spines (Figure 4D), suggesting that intraneuronal expression of tau‐AC by itself did not significantly induce acute synapse loss. However, the number of tau‐AC‐positive dendritic spines was significantly higher than that of tau‐FL‐positive spines (Figure 4D), suggesting that tau‐AC could cause synaptic dysfunction in dendritic spines. Taken together, these findings indicate that tau‐AC‐driven pathological processes in neurons include impaired AIS plasticity and tau mislocalization.

### 2.5 Behavioral Phenotypes and Neuronal Death Induced by Tau‐AC in Mice

**Figure 5:**
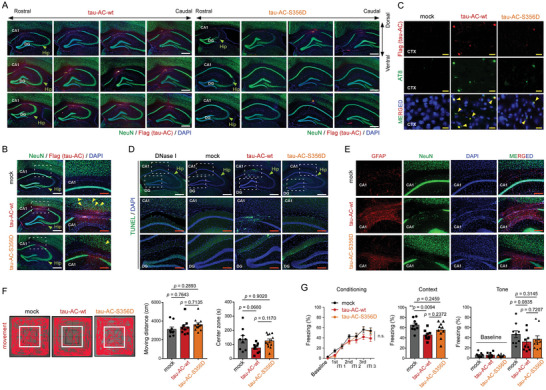
Evaluation of behavioral and biochemical phenotypes of mice hippocampally injected with tau‐AC aggregates. A) Tau‐AC‐wt or ‐S356D (5 µg total/each tau species, after 6 h of in vitro oligomerization) were intra‐hippocampally injected; two months after tau‐AC injection coronal sections were prepared and co‐immunostained with anti‐NeuN and anti‐FLAG antibodies to detect neurons and tau‐AC, respectively. Nuclei were counterstained with DAPI. The ipsilateral hippocampal regions are shown. CA1, cornu ammonis 1; DG, dentate gyrus. B) Representative images of tau‐AC signal in the CA1 area of the ipsilateral hippocampus. Magnified views of boxed areas are shown on right. Yellow arrowheads indicate tau‐AC propagated into the frontal cortex area. C) Two months after the ipsilateral injection of tau‐AC‐wt or tau‐S356D, mice were sacrificed and neurofibrillary tangles in the cortex (CTX) regions were detected in the coronal sections of the brains using anti‐AT‐8 antibodies. Yellow scale bars = 20 µm. D) Apoptotic neurons in the hippocampus were visualized by the terminal deoxynucleotidyl transferase dUTP nick‐end labeling (TUNEL) assay. E) Immunostaining for glial fibrillary acidic protein (GFAP), a glial marker, which is overlapped with the NeuN‐positive signal in the CA1 layer of tau‐AC‐wt‐injected mice. White scale bars = 400 µm, red scale bars = 150 µm. F) One month after stereotaxic injection, mice were subjected to the open‐field test, and their general locomotive activity and anxiety‐like behavior were measured. Representative trace images of the open‐field test (*left*); the center zone is indicated with a white box. Quantification of moving distances (*middle*) and times in the central region (*right*) is shown. Data were analyzed using one‐way ANOVA followed by Tukey post‐hoc test (mock, *n* = 9; tau‐AC‐wt, *n* = 9; tau‐AC‐S356D, *n* = 12; Moving distance, effect of group, F_2,27_ = 1.194, *p* = 0.3185; Center zone, effect of group, F_2,27_ = 3.188, *p* = 0.0572) and presented as the mean ± SEM. G) Freezing behaviors during conditioning, contextual memory retrieval, and tone memory retrieval tasks were evaluated. Two months after tau‐AC injection, mice were trained using three pairs of acoustic tones and electric foot shocks. Freezing behavior at the baseline and in response tones and inter‐tone intervals was monitored (*left*: Conditioning: two‐way repeated measures ANOVA, effect of group, F_2,25_ = 1.979, *p* = 0.1593; mock, *n* = 9; tau‐AC‐wt, *n* = 9; tau‐AC‐S356D, *n* = 10). The next day, freezing behavior was measured in the same conditioning chamber to test contextual fear memory (*middle*: Context, one‐way ANOVA followed by Tukey post‐hoc test, effect of group, F_2,25_ = 5.214, *p = 0.0128). An auditory fear memory was examined in a novel context by measuring freezing at the baseline and in response to tones (*right*: Tone, two‐way ANOVA followed by Tukey post‐hoc test, effect of group, F_2,25_ = 1.291, *p* = 0.2928).

To test whether tau‐AC can recapitulate AD or tauopathy phenotypes in vivo, we stereotaxically injected young (3‐month‐old) non‐transgenic mice with either oligomerized tau‐AC‐wt or pseudo‐phosphorylated tau‐AC‐S356D, into the hippocampus (Figure S6A, Supporting Information). To evaluate tau‐AC‐mediated pathology, we performed immunostaining on the series of coronal sections near the injection site and observed that the tau‐AC‐wt signal was stronger than that of tau‐AC‐S356D (**Figure** **5**A; Figure S6B, Supporting Information). Abundant tau‐AC staining was largely limited to the injection coordinates of the ipsilateral hippocampus; however, a small fraction could also be detected in the anterior and posterior regions. Double staining demonstrated that the high level of tau‐AC signal was associated with the loss of NeuN signal (Figure 5B,C; Figure S6C, Supporting Information). No tau pathology, such as tau filaments, neuronal loss, or inflammation, was observed in control mice. Furthermore, we detected tau filaments in the mouse brains injected with tau‐AC‐wt using anti‐tau antibody AT‐8, which detects the pathological forms of endogenous tau (pSer202/pThr205), but not exogenous tau‐AC (Figure 5C; Figure S6D, Supporting Information). AT‐8‐positive neurons were also largely colocalized with strong FLAG immunoreactivity, which corresponded to tau‐AC aggregates. At the same time, the tau‐AC‐S356D mutant exhibited significantly decreased tau aggregation after the intra‐hippocampal injection (Figure S6D, Supporting Information), suggesting that tau‐AC phosphorylation has an antagonistic effect on self‐nucleation and seeding in vivo as well.

To confirm these findings, we performed histochemical staining using thioflavin S (ThS), which allows the visualization of mature tau tangles. We observed a distinct ThS‐positive signal in the CA1 region of the hippocampus in mice injected with tau‐AC‐wt fibrils, while the ThS‐signal was significantly lower in mice injected with tau‐AC‐S356D (Figure S6E, Supporting Information).^[^ ^28^ ^]^ Using the TUNEL assay, we observed that the high intensity tau‐AC‐positive signal was preferentially overlapped with apoptotic neurons (Figure 5D), providing a possible link between the observed neuronal loss and memory impairment in tau‐AC‐injected mice. In addition to this relatively selective loss of CA1 neurons, the gross morphology of tau‐AC‐injected mice was comparable to that of control mice. Although we did not observe tau‐AC staining in the contralateral, non‐injected hippocampus (Figure S6F, Supporting Information), pathologic tau species could be propagated to the frontal cortex and dendrite gyrus of the ipsilateral hemisphere (Figure 5C; Figure S6G, Supporting Information). Intriguingly, tau‐AC‐wt‐injected mice showed extensive GFAP staining near the CA1 region (Figure 5E; Figure S6H, Supporting Information). These results suggest that tau‐AC, as a potent causal agent of tauopathies, could also induce neuroinflammation and astrogliosis, which are early signals of AD progression and, potentially, protection mechanisms of the brain against pathogenic tau species.

Thereafter, mice were evaluated for their basal locomotor activity and anxiety‐like behavior using an open field test. Mice from both tau‐AC‐wt and ‐S356D groups had moving distances like those of the control mice, indicating that unaffected locomotive functions (Figure 5F). However, we observed that tau‐AC‐wt‐injected mice appeared to spend a longer time in the peripheral region than did controls, suggesting an anxiety‐like behavior, while mice injected with tau‐AC‐S356D showed virtually identical behavior as those injected with tau‐AC‐wt in the open field test (Figure 5F). To evaluate their learning and memory functions, mice were subjected to a conventional fear conditioning test, which is a form of Pavlovian‐associative learning. Two months after the injection, mice were trained with the pairing of acoustic tone and electric shock, with all groups showing similar freezing behaviors in response to baseline, tones, and inter‐tone intervals during the conditioning session (Figure 5G, *left*). The next day, mice were exposed to the same conditioning chamber to test their fear memory, which is dependent on the hippocampus.^[^ ^29^ ^]^ In the contextual fear memory test, tau‐AC‐wt‐injected mice displayed a significantly lower freezing times than those by control mice (Figure 5G, *middle*). At the same time, the mice injected with tau‐AC‐S356D showed freezing times comparable to that shown by control mice. In the auditory fear memory retrieval test, however, the freezing level of tau‐AC‐wt‐injected mice was only mildly reduced compared to those of other groups, suggesting that the tau‐AC‐induced memory deficit appeared to be restricted to the hippocampal test (Figure 5G, *right*). These data collectively suggest that tau‐AC, but not phosphorylated tau‐AC, has a capacity to induce behavioral deficit, including anxiety‐like behavior and hippocampal memory impairment, along with neuropathological phenotypes and neurotoxicity.

## 3 Discussion

Tau aggregation has been implicated in the pathogenesis of the primary and secondary tauopathies, and considerable efforts have been made to identify the underlying molecular mechanisms. Increasing evidence indicates that tau proteins are cleaved by multiple proteases, including calpains, caspases, and endopeptidases, and these tau fragments have pro‐fibrillizing and seeding properties if not properly degraded.^[^ ^30^ ^]^ Numerous studies have also suggested that the R3 and R4 repeats of tau are essential for its pathological role, while the flanking regions may not be required or could even serve a protective role by preventing tau aggregation.^[^ ^31^ ^]^ Using in vitro and in vivo experiments, we demonstrated in this study that the tau residues 306–378 (tau‐AC) of 2N4R were sufficient and essential for the initiation of self‐amplifying cascades. Interestingly, a similar tau fragment was identified as the core of PHFs almost at the same time as tau cDNA was first cloned, and the monoclonal antibodies targeting this fragment had shown that tau truncation could be the seminal event of tauopathy pathogenesis.^[^ ^32^ ^]^

**Figure 6:**
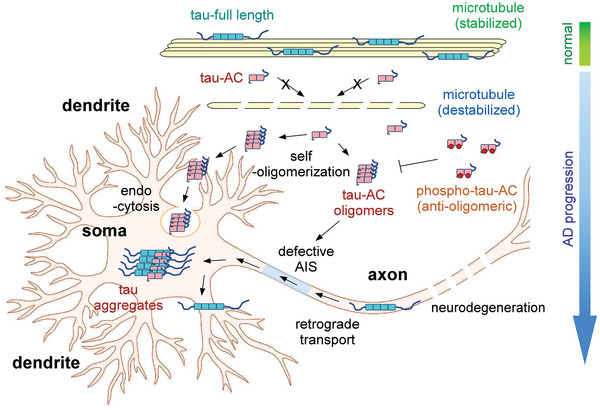
The proposed model of tau‐AC‐induced pathogenesis. Tau‐AC has a weaker binding affinity to microtubules than tau‐FL. During the onset of AD, free (unbound) tau‐AC species spontaneously oligomerize through their N‐terminal residues forming proto‐filaments. Neurons can internalize the oligomeric forms of tau‐AC through endocytosis, leading to endogenous tau aggregation. However, phospho‐mimetic tau‐AC exhibits a drastically reduced aggregation propensity, suggesting that phosphorylation may have anti‐amyloidogenic properties. The pathologic tau species are mislocalized in somatodendrites and dendritic spines due to the perturbed AIS function, ultimately causing excitotoxicity. The clinical significance of this tau fragment in various tauopathies remains to be determined. Furthermore, these findings may pave the way for the development of novel pharmacological approaches designed to inhibit tau filament formation.

We also demonstrated that tau‐AC, which lacks the R1 and R2 repeats of the MBD, was less efficient in binding to tubulin and facilitating microtubule assembly. Thus, the four tandem repeats of the MBD could play distinct roles in the formation of orderly β‐sheets of tau, as suggested by the different compositions of tau aggregation cores in CBD, PiD, and chronic traumatic encephalopathy.^[^ ^5^, ^33^ ^]^ Negatively stained TEM showed that tau‐AC fibrils had a different morphology than that of heparin‐induced tau fibrils, with significantly thicker and shorter filament bundles, which were more likely to become a protofilamental seed and more physiologically related to human AD tau filaments. Therefore, the cleavage of tau‐FL into tau‐AC or corresponding fragments could be the critical event in the pathogenesis of AD (**Figure** **6**). Although it currently remains difficult to identify the exact proteases responsible for the generation of tau fragments, the understanding of cellular conditions that increase amyloidogenic tau‐AC levels is necessary for providing an insight into AD pathogenesis. Recently, TDP43 filaments was identified to be proteolyzed at the post‐fibrillization stage to generate the amyloidogenic core.^[^ ^34^ ^]^ It is conceivable that tau‐AC filaments could undergo the analogous cleavage events. Based on the results of integrative biochemical/biophysical analysis, we propose that the underlying mechanism of tau‐AC aggregation depends on the strong intermolecular interactions between the N‐termini of R3 repeats, the locations of β‐sheet‐forming ^306^VQIVYK^311^ (PHF6) motifs. Compared to that of tau‐FL, tau‐AC is expected to have increased accessibility and flexibility between the PHF6 motifs, initiating the formation of tau proto‐filaments. The majority of mouse lines overexpressing normal tau‐FL are reported to have little signs of obvious tau inclusions or neurodegeneration.^[^ ^35^ ^]^ Therefore, the cleavage of the N‐terminal portion of tau exposes the PHF6 motif as a prerequisite event and onset signal for generating tau nucleates.

As several studies suggested, tau phosphorylation appears to be a complex process regulated by diverse mechanisms and implicated in different pathological processes with either pro‐ or anti‐aggregating properties. We observed that the pseudo‐phosphorylation mutation of S324 or S356 (KxGS motifs in the R3 and R4 repeats, respectively) or both, to Asp significantly shifted the global conformation of tau‐AC from a relatively extended one to a more compact form through electrostatic interactions, shielding N‐termini and limiting the association of PHF6 motifs. Arakhamia et al. reported the polyubiquitylation of multiple Lys residue of tau‐AC in the human AD brain.^[^ ^5d^ ^]^ Because polyubiquitin chains can stabilize β‐strand stacking, we postulated that tau‐AC ubiquitylation, unlike phosphorylation, will further facilitate polymeric tau‐AC formation.^[^ ^3^ ^]^ However, the mechanistic link between tau cleavage and its effect on PTM events (or vice versa) during the course of tauopathies remains to be determined. Since the highly heterogeneous nature of tau aggregates makes it challenging to directly target those structures using conventional small molecules and antibodies, the anti‐aggregation function of tau‐S356 phosphorylation may provide valuable insights for developing more feasible diagnostic and therapeutic tools. Our biochemical studies largely relied on phospho‐mimetic approaches and might not completely replicate the biophysical properties of phosphorylation. Tau‐AC phosphorylation(s) is required to be further characterized using brain samples and validated antibodies. By employing these additional methods, we can obtain a more accurate and reliable assessment of the phosphorylation events occurring on tau‐AC within a pathological context.

Tauopathies progress through the hierarchical stages via neuronal connections. For example, in AD, tau lesions accumulate in a defined spatiotemporal pattern: first, appearing in the transentorhinal cortex, then, spreading to anterior hippocampus and adjacent neocortex, and, finally, invading the primary sensory cortex.^[^ ^36^ ^]^ Missorted tau at somatodendritic compartments can be released into the extra‐neuronal space via secretion or other mechanisms.^[^ ^37^ ^]^ These findings suggest that intra‐neuronal tau proteins, after aggregation, may obtain prion‐ or β‐amyloid‐like properties. Our cell culture experiments demonstrated that tau‐AC aggregates were transcellularly propagated and their uptake was mediated by endocytic processes, providing a plausible mechanism for the in vivo seeding of tau.^[^ ^38^ ^]^ These data also suggest the existence of either a specific extracellular matrix component or even a tau fibril receptor. Since tau seeds in the extracellular space attach to the cell surface by binding to heparan sulfate proteoglycans,^[^ ^39^ ^]^ it is possible that the highly negatively charged proteoglycans may act as receptors (or co‐receptors) for tau aggregates and be involved in subsequent caveolin‐mediated endocytosis. It seems to be rational to hypothesize that the unique biophysical parameters, such as the aggregate size of tau‐AC, contribute to this process.^[^ ^40^ ^]^ PTMs, such as phosphorylation, will add another dimension to this complexity.

Our results suggest neuronal death and neuroinflammation primarily around the site of injection. We also detected propagating tau aggregates in the neighboring cortex regions. However, we could not observe the interlateral tau propagation of tau‐AC in mouse brains, potentially due to a relatively short time frame of our animal experiments (2 months). This also implies that the slow spreading of pathological tau, rather than its aggregate formation, serves as the critical rate‐limiting step of AD progression. The pathologic features of tau‐AC‐injected mouse brains were highly consistent with previous studies reporting a clear correlation between neurofibrillary tangle deposits and cognitive decline.^[^ ^41^ ^]^ This current study also demonstrated that tau‐AC resulted in multiple pathological processes not only in primary neurons (i.e., impaired AIS dynamics and tau trafficking) but also in mice (i.e., defective memory retrieval). We propose that the dysfunctional neuronal excitability (as well as excitotoxicity) in the mouse tauopathy model could be linked to the impaired AIS dynamics in response to tau‐AC aggregates. However, it remains to be further determined whether the defective polarized trafficking of tau was due to altered interactions between tau‐AC and some of AIS elements.

We demonstrated that tau‐AC‐wt injection had a negative effect on the hippocampal memory, with significantly lower freezing times than that in control mice. Unexpectedly, mice injected with tau‐AC‐S356D appeared to show modestly increased freezing times compared to those injected with tau‐AC‐wt; however, the difference was not statistically significant. These findings suggest that we cannot completely rule out the possibility that the phospho‐mimetic mutants still have residual pathogenic effects on hippocampal functions, probably due to the residual self‐aggregation ability. Nevertheless, numerous studies have reported that the phosphorylation of Ser356 in the tau MBD reduced its self‐aggregation propensity,^[^ ^42^ ^]^ consistent with the findings from our biochemical and behavioral assays. In conclusion, we present evidence that tau‐AC functions as a strong causal agent in AD, sufficient to induce the essential features of tauopathies, including self‐fibrillization, spreading, and neurodegeneration. Traditional immunological and biochemical methodologies should be adopted to further establish the pathologic presence of tau‐AC and its etiological roles in human.

## 4 Experimental Section

### Antibodies and Reagents

Antibodies and dilution factors for immunoblotting (IB) and immunofluorescence (IF) staining used in this study were as follows: anti‐β‐actin (A1978, Sigma, 1/10 000), anti‐His (MA1‐21315, Thermo Fisher Scientific; 1/2 000), anti‐FLAG (PA1‐984B, Thermo Fisher Scientific; 1/5 000), anti‐tau‐AC (polyclonal antibody purified from the tau‐AC‐immunized rabbit; 1/2 000; this antibody could detect phospho‐mimetic forms of tau‐AC as well as unphosphorylated tau‐AC.), anti‐tau (clone Tau‐5; Invitrogen; 1/5 000 or 1/300 for IF), anti‐tau Ser202/Thr205 (AT8 clone; MN1020, Thermo Fisher Scientific; 1/100 for IF), anti‐tau Ser356 (ab75603, Abcam; 1/3 000), anti‐MAP2 (AB5622, Millipore; 1/500 for IF), anti‐NeuN (ab177487, Abcam, 1/500 for IHC), anti‐β‐tubulin III (MA1‐19187, Thermo Fisher Scientific; 1/100 for IF), anti‐Rab5 (sc‐46692, Santa Cruz; 1/100 for IF), anti‐Rab7 (sc‐376362, Santa Cruz; 1/100 for IF), anti‐LAMP1 (sc‐17768, Santa Cruz; 1/100 for IF), anti‐GFP (A11122, Thermo Fisher Scientific; 1/300 for IF), mouse anti‐AnkG (N106/65, Neuromab; 1/300 for IF), guinea pig anti‐AnkG (386‐004, Synaptic Systems; 1/300 for IF), anti‐βIV‐spectrin (N393/76, Neuromab; 1/100 for IF), and anti‐EB3 (KT36, ab53360, Abcam, 1/100 for IF). Secondary antibodies for IB (horseradish peroxidase conjugated anti‐mouse IgG and anti‐rabbit IgG antibodies) were acquired from Millipore. The secondary antibodies for IF (goat anti‐rabbit Alexa Fluor 488/594 IgG and goat anti‐mouse Alexa Fluor 488/594/647 IgG) were purchased from Thermo Fisher Scientific. The following endocytosis inhibitors were used: genistein (A2198, Apexbio), chlorpromazine (B1480, Apexbio), and amiloride (B1884, Apexbio). Dulbecco's modified Eagle's medium (DMEM), Roswell Park Memorial Institute (RPMI), and fetal bovine serum (FBS) were purchased from WelGENE.

### Purification of Recombinant Tau Proteins

To purify tau‐FL, tau‐AC, and its mutants, *E. coli* strain Rosetta 2 (DE3) cells (Novagen) were cultured at 37 °C until the OD at 600 nm reached 0.5. Next, 0.5 mm isopropyl‐β‐d‐thiogalactopyranoside (IPTG) was added to induce tau expression and cells were cultured for 4 h at 37 °C. Cells were harvested in a lysis buffer (50 mm phosphate buffer pH 7.0 containing a protease inhibitor cocktail), lysed using sonication, and then heated for 15 min at 85 °C in a water bath. The precipitates were cleared by centrifugation and filtered through a 0.22 µm polypropylene filter. The resulting supernatant was loaded onto a 5 mL HiTrap Mono S column (GE Healthcare Life Sciences) using the ÄKTA FPLC system (GE Healthcare Life Sciences). The column was then washed with five column volumes of washing buffer (50 mm phosphate buffer pH 7.0) and eluted using a NaCl step‐gradient. The eluted proteins were concentrated using Amicon Ultra Centrifugal filters. His‐tagged tau‐AC was purified using a HiTrap TALON crude column. The elution buffer (50 mm phosphate buffer pH 7.0, 300 mm NaCl, 150 mm imidazole) was passed through TALON column to elute tau‐AC protein. Dithiothreitol (DTT) was added to the eluted tau proteins (0.1 mm final concentration) and the proteins were stored at −80 °C. FLAG tags did not change the assembly kinetics of tau‐AC.

### Plasmids and Cloning

For bacterial expression, tau‐FL and tau‐AC constructs were generated using pET28a. The FLAG‐tagged versions of tau‐AC were amplified by adding the FLAG sequence to the reverse primer. Tau‐AC mutants were obtained by PCR‐based site‐directed mutagenesis. To express tau‐AC in mammalian cells, tau‐AC with an HA‐tag at the C‐terminus was subcloned into the pCS6‐SMASh‐CyClinB plasmid vector (from M.H. Glickman, Technion). This plasmid has its own cleavage site between NS3 protease and Tau‐AC‐HA, producing intact tau‐AC with a higher stability, as well as an internal control for tau‐AC expression. To generate EGFP‐tau‐FL and EGFP‐tau‐AC, tau‐FL or tau‐AC were inserted into the EGFP‐tagged tau plasmid vector (Addgene #46 094). A tdTOMATO construct was cloned by transferring tdTOMATO from AAV‐phSYN‐tdTOMATO (Addgene #51 506) to pCAGEN plasmid.

### In Vitro Aggregation Assay

Tau‐FL aggregation was induced using heparin. Briefly, 40 µm of tau‐FL was incubated with 80 µm heparin in the self‐assembly buffer (10 mm HEPES pH 7.4, 100 mm NaCl, 1 mM DTT) on a bench‐top rotating shaker (250 rpm) at 37 °C. Tau‐AC at 50–100 µm concentration self‐aggregated in the assembly buffer with gentle shaking (250 rpm) at 37 °C. To investigate the seeding effect of tau‐AC, 20 μm of tau‐FL and 20 μm of tau‐AC were mixed in the assembly buffer at 37 °C with shaking at 250 rpm.

### Filter Trap Assay, Thioflavin T (ThT) Assay, and Ultracentrifugation Analysis

Before performing filter trap assay and ThT assay, recombinant tau‐AC protein (20 or 40 μm) was incubated in the self‐assembly buffer (10 mm HEPES pH 7.4, 100 mm NaCl, 1 mm DTT) for the designated time point at 37 °C with constant shaking. For filter trap assay, the aggregated tau proteins were mixed with 2× sample buffer (4% SDS, 40 mm EDTA) and then passed through a cellulose acetate membrane (Advantec, 0.2 µm pore size; pre‐activated with 0.1% SDS for 5–10 min), using a 96‐well dot‐blot apparatus (CSL‐D96, Cleaver Scientific) with vacuum aspiration. The membrane was washed twice with 200 µL of 0.1% SDS and trapped proteins were analyzed by immunoblotting. For the ThT assay, 4 µm of tau species were incubated with 50 µm of ThT (Sigma) in a reaction buffer (50 mm glycine‐NaOH, pH 8.5). To evaluate the oligomerization kinetics, fluorescence intensity (excitation at 430 nm and emission at 485 nm) at different reaction time points was measured using a TECAN Infinite M200 fluorometer (Männedorf). After the tau polymerization reaction, samples (100 µL) were centrifuged at 120 000× g for 1 h at 4 °C to separate the supernatant and pellet fractions. All samples were resuspended in SDS‐sample buffer, heated at 85 °C for 10 min, and then subjected to SDS‐PAGE/IB.

### Microtubules Assembly Assay

The ability of recombinant tau‐FL and tau‐AC proteins to accelerate microtubule assembly was evaluated by measuring DAPI fluorescence (excitation at 358 nm, emission at 461 nm) using a TECAN Infinite M200 fluorometer. In a final 100 µL volume reaction, 80 μm tubulin (T240, Cytoskeleton) was mixed with either tau‐FL or tau‐AC (molar ratio tau protein/tubulin = 1/10) in the PEM buffer (80 mm PIPES pH 6.9, 2 mm MgCl_2_, 1 mM GTP, 10 µm DAPI, and 0.5 mm EGTA). Microtubules assembly was determined by monitoring DAPI fluorescence every 60 sec for 1 h at 37 °C. These experiments were performed in triplicate. For tubulin and tau co‐sedimentation, the samples were subjected to ultracentrifugation at 68 000× g for 20 min after the microtubule assembly assay. Pellet and supernatant fractions were analyzed separately using SDS‐PAGE.

### Transmission Electron Microscopy (TEM) Imaging

To obtain the TEM images of tau‐FL and tau‐AC fibrils, a negative staining protocol was used. Uranyl acetate (Sigma–Aldrich) stock solution (0.5% (w/v)) was prepared in HPLC grade water (JT Baker) and filtered through a 0.22 µm disposable syringe filter. Tau proteins (40 µm) were incubated for 72 h and then transferred to a 400‐mesh formvar/carbon Cu(ΙΙ) grid (Electron Microscopy Science). The tau fibril samples (5 µL) were spotted on a Cu(ΙΙ) grid for 3 min at 20 °C and the excess liquid was removed. The grids were washed twice using HPLC‐grade water after removing the samples. Next, each sample was stained with a 5 µL uranyl acetate solution (0.5% w/v) for 1 min and dried for 4 h at 20 °C. TEM images at several magnifications (200 kV, 6 000×, 15 000×, 30 000×, and 60 000×) were obtained using a JEM‐F200 (TFEG) (JEOL Ltd.) field‐emission transmission electron microscope. The length of tau fibrils was measured using the ImageJ software. The fibril length was defined as the end‐to‐end distance of the identifiable negatively stained fibril.

### Tau‐AC Internalization by A549 Cells

A549 cells (Korea Cell Line Bank; #10 185) were cultured in RPMI media supplemented with 10% FBS, 2 mm L‐glutamine, and 100 UmL^−1^ penicillin/streptomycin in a humidified atmosphere with 5% CO_2_ at 37 °C. To form tau‐AC aggregates, recombinant tau‐AC (100 µm) was incubated in the self‐assembly buffer (10 mm HEPES pH 7.4, 100 mm NaCl, 1 mm DTT) for 2 days at 37 °C on a rotating shaker. Next, tau‐AC aggregates were confirmed with immunoblotting and then treated to A549 cells in a 6‐well plate with 1 µm final concentration. After 24 h, cells were trypsinized, re‐plated onto a new 24‐well plate, and allowed to adhere overnight. Immunofluorescence staining was performed to determine the localization of tau‐AC aggregates.

### Seeding of Tau‐AC Aggregates into HEK293 Tau‐P301S FRET Biosensor Cells

Tau‐RD‐P301S‐FRET cells (ATCC #CRL‐3275)^[^ ^23^ ^]^ were cultured in a 24‐well plate in DMEM media until cells reached ≈70% confluency before transducing cells with tau‐AC oligomers. The transduction complexes were generated by combining tau‐AC (at the indicated concentration) with 5 µL of Lipofectamine 2 000 (Invitrogen) in opti‐MEM (Glico) media for a final volume of 100 µL per well. The combined transduction complexes were incubated at room temperature (RT) for 15 min before being added to cells. After 24 h incubation, the effects of oligomeric tau‐AC were analyzed by exciting the cells with a 488 nm laser and captured the fluorescence with a 525/50 filter using an ECHO Revolve R4 microscope.

### Preparation and Analysis of Supernatant and Pellet Fractions from Whole‐Cell Lysates (WCLs)

RIPA‐soluble (supernatant) and ‐insoluble (pellet) fractions were prepared as previously described.^[^ ^43^ ^]^ Briefly, HEK293 cells were washed with ice‐cold PBS three times and then lysed with RIPA buffer. Next, WCLs were centrifuged at 16 000× *g* for 30 min at 4 °C and supernatants were collected. The pellets were washed with RIPA buffer, re‐suspended in RIPA buffer containing 1% SDS, sonicated for 10 s, and heated at 100 °C for 10 min before further analysis.

### Small‐Angle X‐Ray Scattering (SAXS)

Solution SAXS experiments were performed on the 4C SAXS ΙΙ beamline at the Pohang Accelerator Laboratory (Pohang, Republic of Korea). The concentration of tau‐AC was adjusted to 100 μm in a buffer solution (50 mm Tris‐HCl, 6 M Gdn‐HCl, pH 7.4), and the temperature was maintained at 20 °C during exposure to the X‐ray beam. The sample‐to‐detector distance was set at 2 m. During each independent experiment, scattering patterns were recorded for 5 s and measured six times. The Guinier approximation of the SAXS profiles was performed to obtain the radius of gyration (*R*_g_) (Equation 1), where *q* is the scattering vector, and *I*(*q*) is the scattering intensity at *q*.

(1) $$\;\ln\left[I\left(q\right)\right]=\ln\left[I\left(0\right)\right]-R_{g}^{2}/3q^{2}$$

To demonstrate the unfolding of wild‐type and phospho‐mimetic tau‐AC, a Kratky analysis of SAXS profiles (*I*(*q*)·*q* ^2^ as a function of *q*) was performed. The dimensionless Kratky plot was obtained by multiplying the scattering vector *q* by *R*_g_ rather than *q* (*I*(*q*)/*I*(0)·(*q*·*R*_g_)^2^ as a function of *q*·*R*_g_). Modeling of representative structures for each tau‐AC species was performed using EOM analysis from the ATSAS package.^[^ ^44^ ^]^ External pools were used when running a genetic algorithm to optimize ensembles (GAJOE). The size of the ensemble was fixed at 50 curves per ensemble, and repetitions were disallowed. Each distribution was deconvoluted into three fractions with a gaussian distribution (R^2^ values for fitting were 0.99759, 0.99141, 0.99186, and 0.99677 for tau‐AC wt, S324D, S356D, and S324D/S356D, respectively).

### CamSol Analysis

For predicting the solubility of tau‐AC at a pH = 7, the CamSol web server was used (http://www‐vendruscolo.ch.cam.ac.uk/camsolmethod.html).^[^ ^45^ ^]^

### Circular Dichroism (CD) Spectroscopy

CD spectroscopy analysis was performed to investigate the secondary structure of tau‐AC wild‐type and mutant monomers. For that purpose, 200 µL of 40 µm tau‐AC sample was transferred to a quartz cuvette with a 1 mm path length. CD spectra were obtained from 200 to 250 nm at a scan speed of 20 nm min^−1^ performed five times using JASCO J‐815 spectropolarimeter.

### Electrospray Ionization (ESI) Mass Spectrometry (MS) Combined with Ion Mobility Mass Spectrometry (IM‐MS)

IM‐MS was performed in a positive ion mode using a Waters Synapt G2‐Si HDMS quadrupole time‐of‐flight (Q‐TOF) mass spectrometer (Waters). The concentration of tau‐AC samples was adjusted to 5 µm in 20 mm ammonium acetate (pH 6.8), and the proteins were transferred to the gas phase using an ESI source. The capillary voltage was 2.5 kV, and the source temperature was 80 °C. The gas flow rates for helium and drift cells were 180 and 90 mL min^−1^, respectively, which provided a pressure of 3.23 mbar in the drift cell. The CCSs of the tau‐AC conformers were calibrated following the procedure developed by Ruotolo et al.^[^ ^46^ ^]^ and standards described by Bush et al.^[^ ^47^ ^]^

### Molecular Dynamics (MD) Simulations

The external pool (10 000 structures) generation was acquired from the replica‐exchange MD (REMD) simulation with the CHARMM36m force field and the Generalized Born implicit solvation model using the GROMACS software package (version 4.5.5). The initial structure of the wild type tau‐AC monomer was obtained from the tau fibril cryo‐EM structure (PDB ID: 6VH7) from the Protein Data Bank (https://www.rcsb.org). Six replicas (T = 400, 419, 438, 459, 480, and 500 K) were used to obtain an average exchange probability of ≈0.15. Each replica was simulated for 20 ns, and 10 000 MD‐simulated random conformations were extracted from the replicas to perform EOM analysis. The simulation temperatures were generated by the “remd‐temperature‐generator” web server (http://virtualchemistry.org/remd‐temperature‐generator/). MD simulations for dimeric forms were carried out using the GROMACS software package (version 2020.4) and supercomputing resources (KSC‐2021‐RND‐0060) provided by the National Institute of Supercomputing and Network/Korea Institute of Science and Technology Information (KISTI). Based on the 30.4‐µs REMD simulation (400 ns per replica) with the CHARMM36m force field, it was calculated interchain contact probabilities of tau‐AC homodimers. The initial structures were adopted from the replica‐exchange Monte Carlo (REMC) simulations using the CAMPARI simulation package (http://campari.sourceforge.net; ABSINTH model combined with the OPLS‐AA/L parameters implemented in the abs3.2_opls.rpm parameter set). The most extended conformation based on *R*_g_ was selected and duplicated to form a dimer. The simulation protocol and data convergence analysis method were adapted from the protocol described by Man et al.^[^ ^48^ ^]^ Simulations were performed at pH 7 in a cubic TIP3P water box containing 110 mm Na^+^ and Cl^−^ ions. The GROMACS software package (version 2020.4) with the SHAKE algorithm allowed a time step of 2 fs.

For electrostatic interactions, the particle mesh Ewald (PME) method with a cutoff of 1.2 nm was used, while for the van der Waals interactions, the cut‐off was set to 1.2 nm, and a velocity‐rescaling thermostat was employed. The REMD was carried out with 76 replicas from 300 to 400 K, and exchanges between replicas were attempted every 2 ps with an exchange probability of 1.5. The RMSD from the initial structure was merged after 70 ns. The interchain distances between the *β* carbons of each side chain (*α* carbon for glycine) were calculated, and the residues were in contact if the distance between the two designated atoms was smaller than 0.8 nm. A Markov state model (MSM) analysis was performed using the TICAgg. We adopted interatomic distances between the carbon atoms to construct both inter‐ and intra‐chain features. The number of dimensions was fixed at 10, and 300 k‐means clustering centers were represented in the time‐lagged independent component analysis (TICA) space and discretized into three groups. The structures in the figures were modelled using the UCSF Chimera software.

### Primary Rat Cortical and Hippocampal Neurons

For primary neuron cultures, isolated cortical and hippocampal neurons were plated on poly‐D‐lysine‐coated plates and coverslips, respectively, and incubated at 37 °C in a humidified incubator in Neurobasal medium (Invitrogen) supplemented with B‐27 and L‐glutamine (Invitrogen). Tau‐AC aggregates were prepared by incubating tau‐AC (100 µm) at 37 °C for 2 days. At DIV 10, 0.1 μm of tau‐AC aggregates were added to the media and cells were incubated for additional 3 days. At DIV 14, cells were lysed with 1% Triton X‐100 buffer (50 mm Tris‐HCl pH 8.0, 150 mm NaCl, 1% Triton X‐100, 0.1% SDS, 2 mm EDTA, 1× PIC) for IB or fixed with 4% paraformaldehyde (PFA) for IF. For overexpression experiments, tau‐AC and its phospho‐mimetic mutants were first cloned into the FUGW lentiviral vector. Lentiviral particles were produced by co‐transfection of vesicular stomatitis virus glycoprotein, Δ8.9, and the lentiviral vector in the packaging cell line HEK293T. After 60 h of incubation, the media containing the lentiviral particles was harvested, centrifuged to remove cellular debris, aliquoted, and stored at −80 °C. Primary cultured neurons were infected with lentiviral particles expressing tau‐AC and its mutants at DIV 6. At DIV 14, cells were lysed for IB or fixed for IF.

### Chronic Depolarization, Polarized Trafficking, and Dendritic Spines

At DIV 10, primary hippocampal neurons were transfected with EGFP, EGFP‐tau‐FL, and EGFP‐tau‐AC. After 48 h, the cultured neurons were depolarized by raising the KCl concentration of the medium from 5 to 15 mm for additional 48 h; 10 mm NaCl was added to the control cells. For IF, cells were fixed in 4% PFA in PBS for 20 min and then permeabilized with 0.25% Triton X‐100 for 5 min. Next, sections were blocked for 1 h, incubated, first, with primary antibodies at 4 °C overnight, and, subsequently, with secondary antibodies for 1 h at RT. Fluorescent images were acquired using Zeiss LSM800 (40× lens, 512×512 pixels, and merging 0.4 µm Z‐stacks by orthogonal projection) or ECHO Revolved Fluorescence microscope. The AIS length was measured and analyzed using the MATLAB Measurements and MathWorks program. The AIS start, maximum, and end positions were determined as previously described.^[^ ^24^ ^]^ The data for AIS positions were analyzed by two‐way ANOVA with Turkey multiple comparison test. AIS length was defined as the length between the start and end points and data were analyzed using one‐way ANOVA with Turkey multiple comparison tests. For polarized trafficking, plasmids expressing EGFP‐tau‐FL or EGFP‐tau‐AC were co‐transfected with tdTOMATO (Addgene plasmid #51 506) into DIV 13 hippocampal neurons for 24 h and AnkG immunostaining was performed as described above. The axon‐to‐dendrite ratio was calculated as [(axonal tau fluorescence/dendritic tau fluorescence)/(axonal area by tdTOMATO)/dendritic area by tdTOMATO].^[^ ^49^ ^]^ The data were analyzed using Student's *t*‐test and the GraphPad Prism 6 software. The area of the dendritic spine was measured by ImageJ plug‐in SpineJ.^[^ ^50^ ^]^ Dendrites were counted as tau‐positive when EGFP‐tau signals occupied more than 50% of tdTOMATO signals in total dendritic spine areas.

### Stereotaxic Injection

Nine‐week‐old male C57BL6/J wild‐type mice (N = 10) were injected with tau‐AC aggregates. Briefly, mice were deeply anesthetized using the intraperitoneal injection of a Zoletil/Rompun mixture (30 and 10 mg kg^−1^, respectively), fixed on a stereotaxic apparatus (Kopf instruments), and 1 µL of tau‐AC aggregates (wt and S356D; both proteins (500 μm) were incubated for 6 h at 37 °C with gentle agitation) was unilaterally stereotaxically injected into the hippocampal CA1 region using a 30‐gauge syringe (Hamilton). The coordinates were as follows: AP: −1.8 mm from the bregma, ML: +1.0 mm from the bregma, DV: −1.7 mm from the skull. The mock group underwent the same surgical procedures without the injection step. Experiments were conducted in accordance with the guidelines approved by the Institutional Animal Care and Use Committee of Seoul National University (IACUC #: SNU 171220‐2‐5).

### Behavior Tests

Open‐field testing was used to measure the overall locomotor activity, novelty seeking, and anxiety levels of mice. In a white 32 cm × 32 cm × 40 cm arena, mice were placed at the center (20 cm × 20 cm) and tracked for 15 min using a computerized video tracking system. Anxiolytic and exploratory behaviors were characterized by exploring the center of the field rather than staying in the corner. Data were analyzed using the Ethovision 14 software (Noldus). Fear conditioning was performed as previously described with slight modifications.^[^ ^51^ ^]^ Briefly, on day one, after exploring a fear conditioning chamber (Coulbourn Instruments) for 3 minutes, mice received three pairs of electrical footshocks (0.5 mA, 2 s) and a conditioned acoustic stimulus (CS, 80 dB, 2800 Hz, 30 s), which were co‐terminated. The CS was delivered with 30 s‐inter‐tone interval. On day two, the mice were placed in the same fear conditioning chamber for 120 s. On the same day, the mice were placed in a novel cylinder chamber and received the CS for 120 s after 120 s baseline period. During all sessions, freezing behavior for at least 1 s was automatically measured using the Freeze Frame software (ActiMetrics, IL, USA).

### Perfusion

After anesthesia, mice were perfused with PBS, followed by 4% PFA. Brains were dissected, post‐fixed in 4% PFA overnight at 4 °C, and then transferred to a 30% (w/v) sucrose solution. Brains were sectioned into 30‐µm coronal sections using a cryotome and stored in a cryoprotectant solution (25% glycerol, 25% ethylene glycol, 20 mm phosphate buffer pH 7.4) at −20 °C.

### TUNEL Assay

Apoptotic cells were identified using a TUNEL assay following the manufacturer's protocol (TUNEL Andy Fluor^TM^ 488 Apoptosis Detection Kit; ABP Biosciences) with minor modifications. Briefly, brain sections were washed twice in PBS, permeabilized with 0.2% Triton X‐100/PBS for 30 min at room temperature, and then incubated in a TUNEL solution for 2 h in a humid chamber at 37 °C. Next, samples were washed with 3% BSA in PBS and then placed in the staining solution for 1 h at RT.

### Statistical Analysis

Statistical significance of differences between various groups was determined by Student's *t*‐test or one‐way ANOVA followed by the Bonferroni *post‐hoc* test for most data. All experiments were performed in triplicates and data are presented as the mean ± standard deviation (SD). Differences were considered significant when *p*‐values were less than 0.05.

## Conflict of Interest

The authors declare no conflict of interest.

## Author Contributions

L.T.H.L.L., J.L., D.I., S.P., K.‐D.H., contributed equally to this work. L.L. and J.L. carried out most of the biochemical experiments. D.I. and H.I.K. performed the biophysical analysis. S.P. and Y.H.S. conducted the AIS‐related experiments. K.D.H. and Y.‐S.L. performed the mouse behavior analysis. Y.J. and J.H.L. prepared the recombinant proteins and primary neurons. M.J.L. Y.‐S.L., Y.H.S, and H.I.K. prepared and revised the manuscript. M.J.L. is responsible for the overall design of the study and manuscript preparation. All the authors reviewed the manuscript and approved the final version.

## Supporting information

Supporting Information

## Data Availability

The data that support the findings of this study are available in the supplementary material of this article.
