# Supplementary material for: Self‐Aggregating Tau Fragments Recapitulate Pathologic Phenotypes and Neurotoxicity of Alzheimer's Disease in Mice
Source: Adv Sci (Weinh). 2023 Aug 18;10(29):2302035. doi: 10.1002/advs.202302035 (PMC10582461; doi:10.1002/advs.202302035)
Supplement: Supplementary file 1 — Supporting Information [file ADVS-10-2302035-s001.pdf]

ADVANCED SCIENCE
Open Access

Supporting Information

for *Adv. Sci.*, DOI 10.1002/advs.202302035

Self-Aggregating Tau Fragments Recapitulate Pathologic Phenotypes and Neurotoxicity of Alzheimer's Disease in Mice

Ly Thi Huong Luu Le, Jeeyoung Lee, Dongjoon Im, Sunha Park, Kyoung-Doo Hwang, Jung Hoon Lee, Yanxialei Jiang, Yong-Seok Lee, Young Ho Suh*, Hugh I. Kim* and Min Jae Lee**

Self-aggregating Tau Fragments Recapitulate Pathologic Phenotype and Neurotoxicity of Alzheimer's Disease in Mice

Ly Thi Huong Luu Le, Jeeyoung Lee, Dongjoon Im, Sunha Park, Kyoung-Doo Hwang, Jung Hoon Lee, Yanxialei Jiang, Yong-Seok Lee, Young Ho Suh, Hugh I. Kim, and Min Jae Lee

Table of Contents

Supporting figures and figure legends

Figure S1. Seeding effect of tau-AC on tau-FL aggregation *in vitro*…………………………..... S2
Figure S2. Biochemical analysis of tau-AC and its phosphor-mimetics…………………..…….. S4
Figure S3. Biophysical analysis of tau-AC and its phosphor-mimetics……………….……... S5
Figure S4. Reduced aggregation-propensity of tau-AC lacking the N-terminal PHF6 motif and phosphor-mimetic tau-AC…………………………………………………………………… S7
Figure S5. Internalization of tau-AC aggregates into cells…………………………………… S8
Figure S6. The effect of stereotaxically injected tau-AC-wt or -S356D on the behavioral and biochemical phenotypes of mice……………....……………………………………………… S10

Supporting figures and figure legends

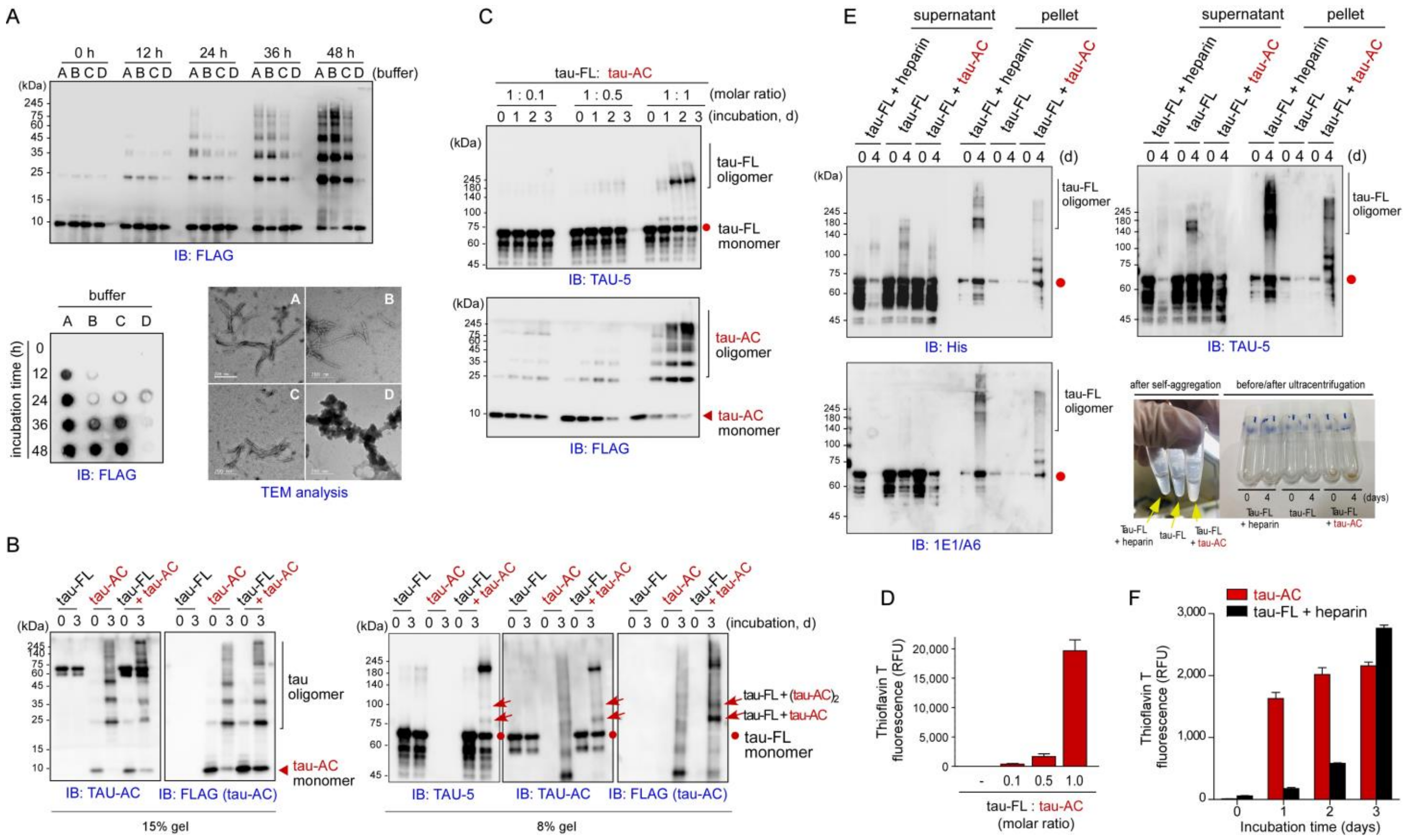

Figure S1. Seeding effect of tau-AC on tau-FL aggregation *in vitro*. (**A**) Tau-AC self-aggregation experiments were performed as described in Fig. 1A using four different buffer (Buffer A, 10 mM HEPES, 100 mM NaCl, 1 mM DTT, pH 7.4; Buffer B, 10 mM phosphate buffer, 10 mM DTT, pH 7.4; Buffer C, 10 mM phosphate buffer, 100 mM NaCl, 10 mM DTT, pH 7.4; Buffer D, 10 mM phosphate buffer, 200 mM $MgCl_2$, 10 mM DTT, pH 7.4). The samples were analyzed with the following analyses. (*top*) Non-reducing SDS-PAGE followed with anti-FLAG immunoblotting (IB). (*bottom, left*) Filter-trap analysis/IB using anti-FLAG antibodies. (*bottom, right*) Transmission electron microscopy (TEM) analysis using tau-AC samples incubated for 48 h. . (**B**) Tau-FL (20 μM) and tau-AC^{FLAG} (20 μM) were incubated at 37°C with mild agitation. After 3 days, samples were subjected to non-reducing SDS-PAGE and immunoblotting (IB) using

anti-FLAG, TAU-5, and TAU-AC antibodies. Representative blot from three experiments is shown. (**C**) Samples were incubated as described in (**A**) using different reactant ratios and incubation times as indicated. (**D**) Tau-FL was incubated with tau-AC (molar ratios 0.1, 0.5, or 1.0) for 2 days, and thioflavin T (ThT) fluorescence intensity was measured at 485 nm. (**E**) Tau-FL (20 μM) proteins were incubated with either heparin (80 μM) or tau-AC (20 μM) for 4 days and then subjected to ultracentrifugation at 120,000× *g* for 1 h. The supernatant and pellet fractions were collected and analyzed by SDS-PAGE/IB with antibodies as indicated. (**F**) ThT assays were performed using tau-AC (20 μM) only and Tau-FL (20 μM) combined with heparin (80 μM) for the indicated time periods. All immunoblots shown are representative from two or three independent experiments. These data supplement Figure 1.

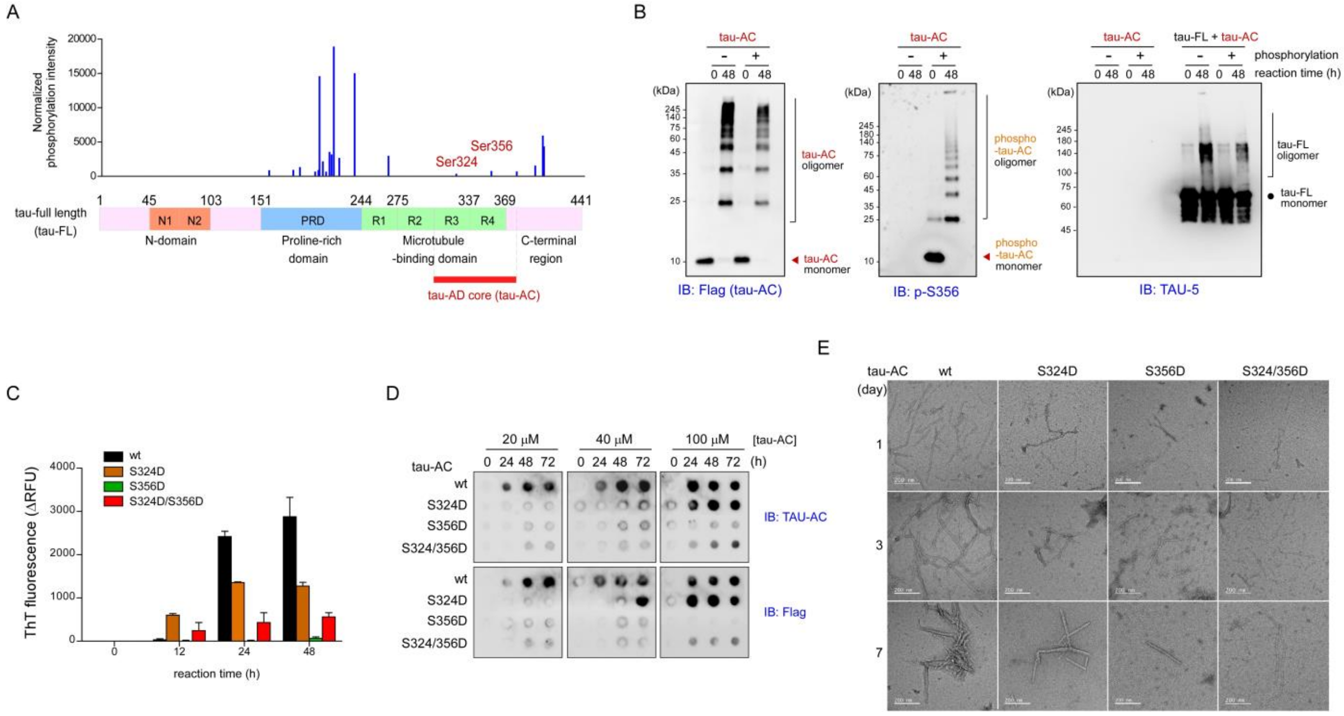

Figure S2. Biochemical analysis of tau-AC and its phospho-mimetics. (**A**) Recombinant His-tau (0.5 μg) was phosphorylated *in vitro* by GSK3β (1 μg with 1 mM ATP) for 4 h and then subjected to MS analysis. Even though there are numerous phosphorylation sites in the proline-rich domain, only Ser324 and Ser356 in the tau-AC region are identified to be phosphorylated. (**B**) *In vitro* phosphorylation reactions were performed by incubating tau-AC and GSK3β at 30°C for 16 h. Next, phosphorylated tau-AC (20 μM) was incubated with tau-FL (20 μM) for 2 days at 37°C with mild agitation and samples were analyzed by IB using antibodies as indicated. Representative blot from two experiments is shown. (**C**) The ThT assay was performed using 4 μM of tau-AC-wt and its phospho-mimetic mutants (S324D, S356D, and SDSD double-mutants); samples were incubated for the indicated time periods. (**D**) Filter-trap assays using recombinant tau-AC species were performed with concentrations and incubation times as indicated. Trapped tau fibrils were detected by IB using indicated antibodies. (**E**) Representative TEM images of negatively stained tau filaments (tau-AC-wt, -S324D, -S356D, and -S324D/S356D). Recombinant tau-AC and its mutants (20 μM) were incubated for 1, 3, or 7 days at 37°C. Scale bars = 200 nm.

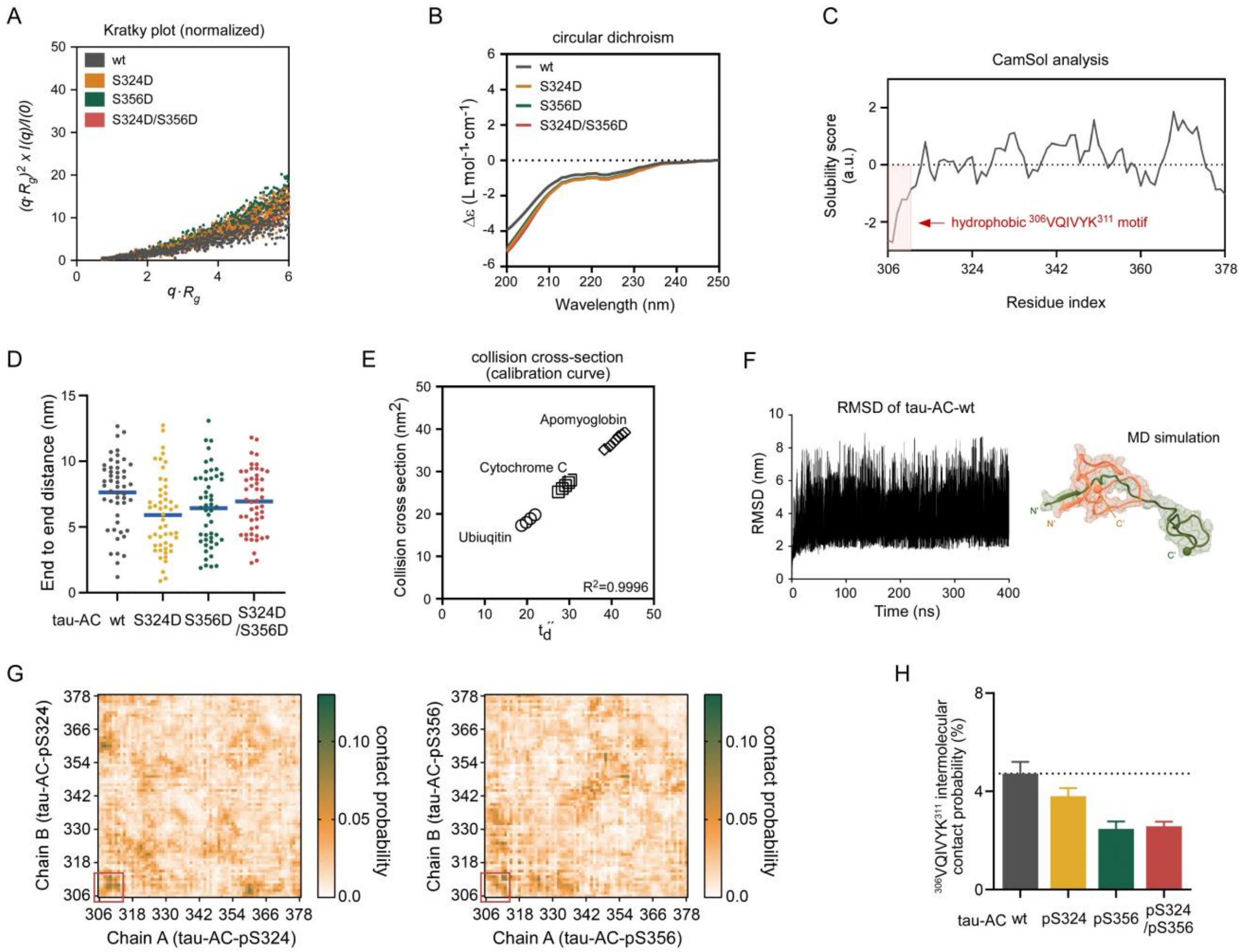

Figure S3. Biophysical analysis of tau-AC and its phosphor-mimetics. (**A**) Kratky analysis of tau-AC wild-type (wt), its single phospho-mimetic mutants (S324D and S356D), and double phospho-mimetic mutants (S324D/S356D) was performed using small-angle X-ray scattering profiles. (**B**) Circular dichroism spectra of tau-AC-wt and phospho-mimetic mutants. (**C**) CamSol solubility profile of tau-AC at neutral pH (pH = 7). The PHF6 residues (amino acids 306 – 311) located at the N-terminus show poor solubility. (**D**) Structural conversion of tau-AC-wt and its mutants observed in 50 ensemble structures obtained from EOM analysis. End-to-end distance calculations (7.63 ± 0.37 nm for the wild-type and 5.91 ± 0.41, 6.43 ± 0.40, and 6.95 ± 0.34 nm

for the S324D and S356D and S324D/S356D variants, respectively). (**E**) The collision cross-section calibration curves using standard proteins with known CCS values, such as ubiquitin, cytochrome C, and apomyoglobin (R^2 > 0.999). (**F**) Root-mean-square deviation (RMSD) data from the MD simulation (*top*) and the representative structures (*bottom*) of tau-AC-wt homodimers. The RMSD from the initial structure was merged after 70 ns. Two chains in the structure are depicted in different colors (orange and green) and the C-terminal end of each chain is resented as a sphere. (**G**) Interchain contact probability maps of single phosphorylated tau-AC at S324 and S356 (tau-AC-pS324 and –pS356, respectively). Identical calculations were performed as in Fig. 2H. Red boxes indicate the ^{306}VQIVYK^{311} hexapeptides located at the N-termini of tau-AC. (**H**) Averaged intermolecular contact probability between the ^{306}VQIVYK^{311} hexapeptides of tau-AC-wt and its phosphorylated forms (4.72 ± 0.47% in the wild-type and 3.80 ± 0.33%, 2.47 ± 0.30%, and 2.58 ± 0.18% in the pS324, pS356, and pS324/pS356, respectively). The error bars represent the standard error of mean. These data supplement Figure 2.

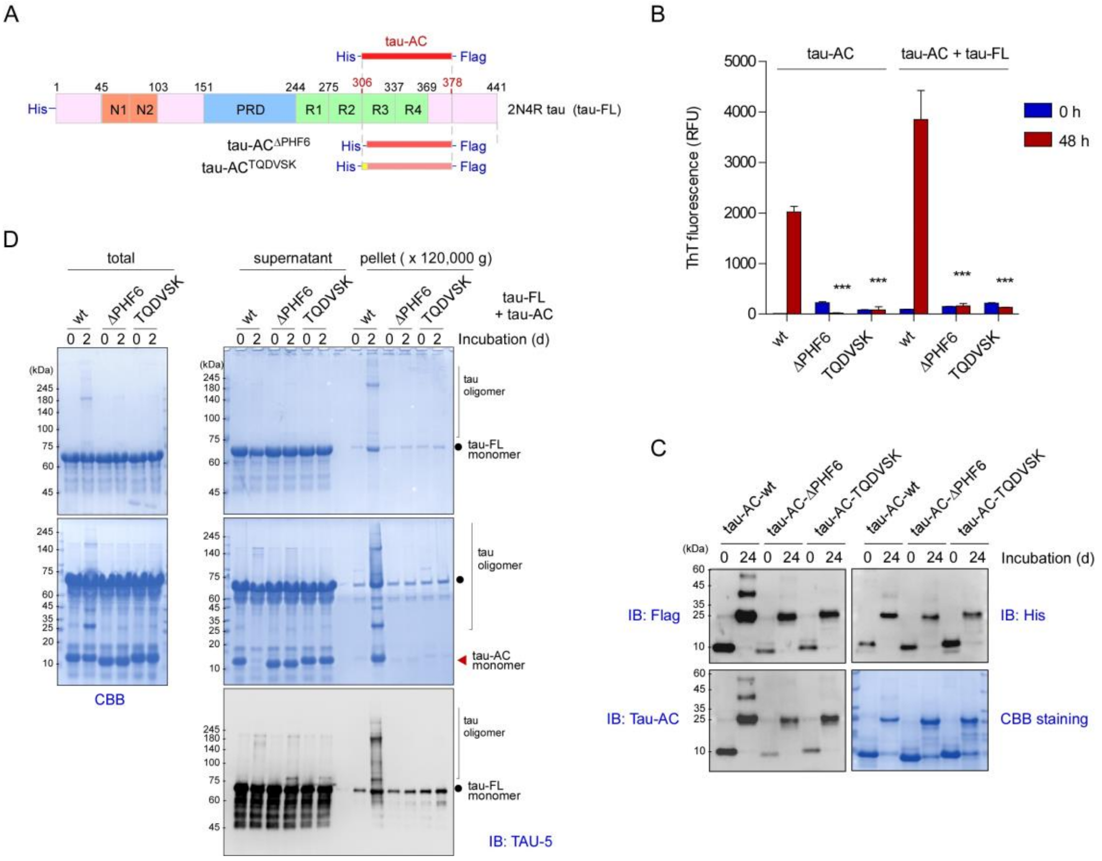

Figure S4. Reduced aggregation-propensity of tau-AC lacking the N-terminal PHF6 motif and phospho-mimetic tau-AC. (**A**) Schematic diagram of tau-AC mutants where the PHF6 motif was deleted (tau-AC-ΔPHF6) or substituted with hydrophilic residues (tau-AC-TQDVSK). (**B**) Markedly reduced ThT signal intensities of the PHF6 mutants compared to that of tau-AC-wt. Both tau-AC only and tau-AC/tau-FL co-incubation conditions were evaluated using the mutant species as described in Fig. 1E. Error bars represent standard errors of the mean. RFU, relative fluorescence unit. ***$p < 0.001$ ($n = 3$, one-way ANOVA). (**C**) Loss of self-aggregation propensity of tau-AC mutants lacking the hydrophobic PHF6 motif. Tau-AC-wt (20 μM each) were incubated with either tau-AC-ΔPHF6 or -TQDVSK for self-oligomerization for 24 h. The sampled were subjectd to non-reducing SDS-PAGE/IB with indicated antibodies and Coomassie Brilliant Blue (CBB) staining. Representative blots from two independent experiments. (**D**) As in (C), except that tau-AC species were co-incubated with tau-FL (20 μM), and the samples were separated into supernatant and pellet fractions with ultracentrifugation (at 68,000× g for 20 min) before SDS-PAGE/IB analyses.

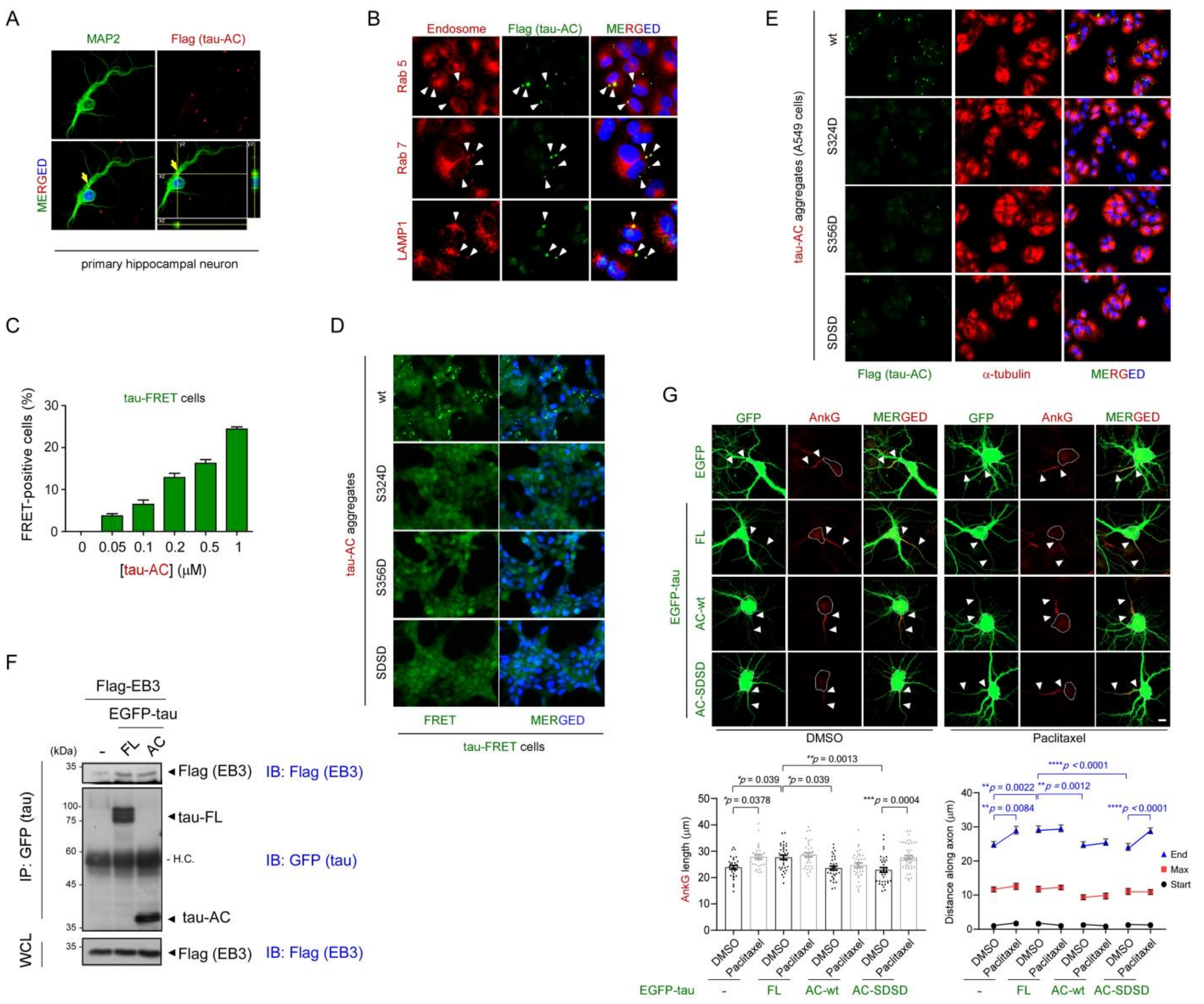

Figure S5. Cellular uptake of tau-AC aggregates. (**A**) Day 10 *in vitro* (DIV 10) primary rat hippocampal neurons were incubated with oligomerized tau-AC (0.1 μM) for 3 days, vigorously washed three times, and then co-immunostained using anti-MAP2 (for differentiated neuron) and anti-FLAG (for tau-AC) antibodies. Nuclei were counterstained with DAPI. Yellow arrows indicate intraneuronal tau-AC. (**B**) A549 cells were treated with 1 μM of tau-AC aggregates for 24 h, trypsinized, replated, and then immunostained with anti-FLAG (tau-AC), anti-Rab5 (early

endosomes), anti-Rab7 (late endosomes), or anti-LAMP1 (lysosomes) antibodies. Arrowheads point to tau-AC colocalized with the components of the endosomal-lysosomal pathway. (**C**) HEK293 tau-P301S biosensor cells were treated with oligomeric forms of tau-AC-wt. After 24 h, cells were fixed and FRET signals were measured. FRET-positive signals were not observed in cells treated with tau-AC monomers. (**D**) Experiments were performed as described in (**C**) using tau-AC-wt and its phospho-mimetic mutants (tau-AC-S324D, -S356D, and -SDSD) to obtain the FRET images. Before added to the cells, tau species were subjected to *in vitro* oligomerization reactions for 48 h and the same amount (1 μM total) of tau were incubated to the cells for another 24 h. (**E**) Representative images of A549 cells incubated with tau-AC-wt and its phospho-mimetic mutants for 24 h. Internalized tau-AC species were detected by immunofluorescence using an anti-FLAG antibody. These data supplement Figure 3. (**F**) Direct interaction between End-binding protein 3 (EB3) and tau-AC. FLAG-tagged EB3 and EGFP-tagged tau-FL or tau-AC were transiently overexpressed in HEK 293T cells for 2 days. Whole-cell lysates were subjected to immunoprecipitation using anti-GFP antibodies, followed by IB as indicated. H.C., heavy chain. **(G)** Tau-AC-SDSD mutant restores AIS structural plasticity. DIV 14 primary hippocampal neurons expressing EGFP (vec), EGFP-tagged tau-FL, tau-AC-wt, or tau-AC-S324D/S356D (SDSD) were treated with 1 nM paclitaxel, a microtubule-stabilizing agent, for 3 h. AIS localization and lengths were analyzed as described in Fig. 4A. White arrowheads represent the start and end points of the AIS. Scale bar, 10 μm. Distances along the axon and AIS lengths were analyzed using two-way ANOVA and one-way ANOVA, respectively, followed by Tukey multiple comparison test (N = 3).

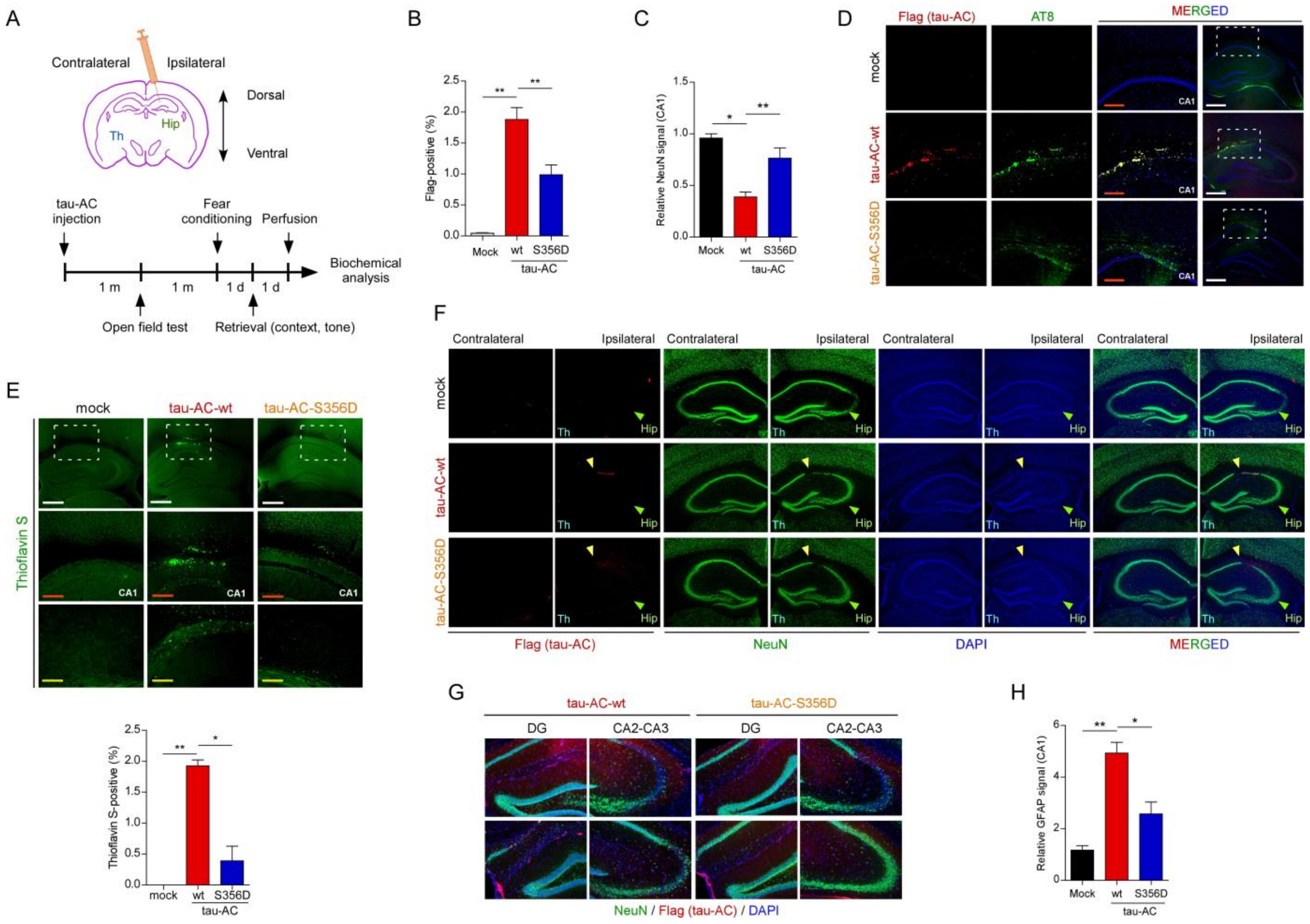

Figure S6. The effect of stereotaxically injected tau-AC-wt or -S356D on the behavioral and biochemical phenotypes of mice. (**A**) Experimental design of the study. A unilateral injection of either tau-AC-wt or -S356D mutant (a total 5 μg proteins after *in vitro* oligomerization reactions for 48 h) was administered into the hippocampus of 9-week old wild-type mice. Hip, hippocampus; Th, thalamus. (**B**) Quantitation of Figure 5B. The intensity of FLAG-positive signals (tau-AC) was measured and plotted as the mean ± SD (mock, n = 9; tau-AC-wt, n = 9; tau-AC-S356D, n = 10). **p* < 0.05, ***p* < 0.01, ****p* < 0.001 (one-way ANOVA followed by the Bonferroni post-hoc test). (**C**) Quantification of NeuN intensity in the CA1 region. Data were analyzed using one-way ANOVA with Bonferroni's multiple comparison test (n =3/group). (**D**) Lower magnification

images of Fig. 5C. (**E**) (*upper*) Neurofibrillary tangles in the hippocampus and CA1 regions were detected using thioflavin S staining. White, red, and yellow scale bars = 400 μm, 200 μm, and 100 μm, respectively. (*lower*) Thioflavin intensity was measured and plotted as the mean ± SD (one-way ANOVA followed by the Bonferroni post-hoc test, N = 3). (**F**) Experiments were performed as described in Fig. 5A; lower magnifications with the contralateral slides are shown. Hip, hippocampus (green arrowheads); Th, thalamus. Yellow arrowheads indicate CA1 regions with neurotoxicity, which correspond to tau-AC signal colocalization. (**G**) The dendrite gyrus (DG) and CA2-CA3 regions were stained for NeuN (neuron marker) and FLAG (injected tau-AC). **(H)** GFAP intensity in the CA1 region was quantified and analyzed using one-way ANOVA with Bonferroni's multiple comparison test (n=3/group).
